# Projected Carbon Dioxide to Increase Grass Pollen and Allergen Exposure Despite Higher Ozone Levels

**DOI:** 10.1371/journal.pone.0111712

**Published:** 2014-11-05

**Authors:** Jennifer M. Albertine, William J. Manning, Michelle DaCosta, Kristina A. Stinson, Michael L. Muilenberg, Christine A. Rogers

**Affiliations:** 1 Harvard Forest, Harvard University, Petersham, MA 01366, United States of America; 2 Stockbridge School of Agriculture, University of Massachusetts, Amherst, MA 01003, United States of America; 3 Department of Environmental Conservation, University of Massachusetts, Amherst, MA 01003, United States of America; 4 Environmental Health Sciences, School of Public Health and Health Sciences, University of Massachusetts, Amherst, MA 01003, United States of America; Institute of Botany, Chinese Academy of Sciences, China

## Abstract

One expected effect of climate change on human health is increasing allergic and asthmatic symptoms through changes in pollen biology. Allergic diseases have a large impact on human health globally, with 10–30% of the population affected by allergic rhinitis and more than 300 million affected by asthma. Pollen from grass species, which are highly allergenic and occur worldwide, elicits allergic responses in 20% of the general population and 40% of atopic individuals. Here we examine the effects of elevated levels of two greenhouse gases, carbon dioxide (CO_2_), a growth and reproductive stimulator of plants, and ozone (O_3_), a repressor, on pollen and allergen production in Timothy grass (*Phleum pratense* L.). We conducted a fully factorial experiment in which plants were grown at ambient and/or elevated levels of O_3_ and CO_2_, to simulate present and projected levels of both gases and their potential interactive effects. We captured and counted pollen from flowers in each treatment and assayed for concentrations of the allergen protein, Phl p 5. We found that elevated levels of CO_2_ increased the amount of grass pollen produced by ∼50% per flower, regardless of O_3_ levels. Elevated O_3_ significantly reduced the Phl p 5 content of the pollen but the net effect of rising pollen numbers with elevated CO_2_ indicate increased allergen exposure under elevated levels of both greenhouse gases. Using quantitative estimates of increased pollen production and number of flowering plants per treatment, we estimated that airborne grass pollen concentrations will increase in the future up to ∼200%. Due to the widespread existence of grasses and the particular importance of *P. pratense* in eliciting allergic responses, our findings provide evidence for significant impacts on human health worldwide as a result of future climate change.

## Introduction

One expected effect of climate change on human health is increasing allergic and asthmatic symptoms through changes in pollen biology [1]–[5]. Allergic diseases have a large impact on human health globally, with 10–30% of the population affected by allergic rhinitis and more than 300 million affected by asthma [6]. Pollen from grass species, which are highly allergenic and occur worldwide, elicits allergic responses in 20% of the general population and 40% of atopic individuals [7].

Climate change is noticeably affecting plant, animal and human systems and is anticipated to have large impacts on human health [4], [5], [8], [9]. A major concern is that wind-borne pollen, a primary cause of allergic rhinitis, may change in timing, amount, and allergenicity with future climate change, and may increase both the symptom severity and number of people affected [1], [3], [5], [9], [10], [11]. This concern is particularly relevant for grasses, which are widely distributed over the globe and affect a large number of sensitized individuals [1], [4], [12]. In fact, peaks in atmospheric grass pollen have been directly correlated to ambulance calls by patients under respiratory stress [13] and ER visits for asthma and wheeze [14].

Here we examine the effects of elevated levels of two greenhouse gases, carbon dioxide (CO_2_), a growth and reproductive stimulator of plants, and ozone (O_3_), a repressor, on pollen and allergen production in Timothy grass (*Phleum pratense* L.).

Atmospheric CO_2_ levels resulting from anthropogenic sources, a major driver of climate change, are expected to rise from around 400 ppm currently to 730–1020 ppm by the year 2100 [15]. Likewise, tropospheric O_3_ has been predicted to rise from current background levels of about 30–40 ppb to 42–84 ppb by 2100 [15], [16]. These greenhouse gases have been shown to affect plant growth in contrasting ways. Carbon dioxide has been well characterized as stimulating plant growth through increased photosynthetic carbon assimilation [17]. Ozone has been shown to decrease growth due to oxidative damage of photosynthetic components [18]. Together these two gases act antagonistically on plants, the degree to which depends highly on plant species and other environmental factors [18].

The effects of elevated CO_2_ on the production and allergen content of grass pollen have not been examined to date [4], although increases in pollen production 55–90% have been reported in other allergenic plants, such as ragweed (*Ambrosia artemisiifolia* L.), at elevated CO_2_ [9], [19], [20]. Elevated CO_2_ has also been shown to increase the reproductive output of ragweed populations through increasing the reproduction of subordinate plants in a competitive stand [21]. In a meta-analysis, C_3_ and C_4_ grass species demonstrated 44% and 33% growth stimulation, respectively, in response to elevated CO_2_ [22]. It is therefore likely that elevated CO_2_ could also increase pollen output in grasses_._ *In vivo* exposure to O_3_ has been shown to reduce amount of viable pollen by preventing pollen maturation through reduced anther starch content in perennial rye grass (*Lolium perenne* L.*)* and increase the group 5 allergen content of the pollen [23], [24]. Ozone has also been shown to decrease pollen viability of *in vitro* exposed Timothy grass pollen through disruption of the cell membrane; however, this disruption could increase allergen exposure by releasing cytoplasmic allergen-containing granules from within pollen [25], [26]. Finally, allergenicity has been found to decrease in Timothy grass pollen from reduced IgE recognition due to mechanical damage and post translational modifications when pollen was exposed *in vitro* to the mixture of air pollutants: ozone, sulfur dioxide, and nitrogen dioxide [26]. While these studies suggest that there could be a reduction in pollen viability and change in allergen quality at projected O_3_ levels, it is not clear how these effects would interact with expected stimulatory effects of CO_2_ on growth and reproduction in grasses.

Timothy grass (*Phleum pratense* L.) is a widespread perennial C_3_ grass species used in agriculture and found growing naturally throughout temperate zones of North America and Europe. It produces a single inflorescence per plant with abundant amounts of easily aerosolized pollen, and is a major cause of early summer allergies. The allergens in Timothy grass pollen are similar to, and cross-reactive with, allergens from many other grass taxa; hence Timothy grass pollen extracts are often used in skin testing for broadly diagnosing grass allergy [7], [12]. The processes that affect these proteins in Timothy grass may also be representative of responses in other grass taxa.

In this study we determined the interactive effects of CO_2_ and O_3_ at current levels (400 ppm CO_2,_ 30 ppb O_3_) and at projected elevated levels (800 ppm CO_2,_ 80 ppb O_3_) in a full factorial design, on both the amount of pollen produced and the concentration of Phl p 5 protein, the major allergen in Timothy grass pollen. Plant exposure to gas treatments was entirely *in vivo* in order to assess whole plant responses. On each plant we measured: number of pollen grains per inflorescence, concentration of Phl p 5 per inflorescence and per pollen grain, inflorescence weight, inflorescence length, and number of inflorescences produced. Using generalized linear model approaches, we examined the effects of the factorial treatments on each parameter, and on the relationships between parameters.

## Methods

Seeds of *Phleum pratense* L. var. CLIMAX were sown in 10.5 cm×10.5 cm×35 cm pots (Treepots, Hummert International, Missouri, USA) at the rate of 20 seeds per pot using MM 200 growing medium (SunGro, Washington, USA). At seeding, equal numbers of pots were placed per treatment in continuously-stirred tank reactor (CSTRs) chambers [27] and plants were allowed to germinate and grow through maturity. Grass was fertilized weekly with a dilute concentration of Peat Light Special (1/3 concentration; 15-16-17; Peter’s Professional; Scotts, Ohio USA) and watered as needed. Plants were exposed to natural light since chambers were located in a single greenhouse, however light was also supplemented with 400W metal Halide lights (Metal Arc, Sylvania, Massachusetts, USA) which were on 12- hours/day for the first five weeks, then on for 16-hours/day for the remainder of the experiment. Greenhouse temperatures ranged from 15.5°C–26°C. Chambers were monitored for temperature and relative humidity levels using Hoboware data loggers (Onset Computer Corp; Massachusetts, USA).

Eight CSTRs [27] chambers were assigned to factorial treatments with either 30 ppb or 80 ppb ozone and either 400 ppm or 800 ppm carbon dioxide, to simulate present and future projected levels of both gases, respectively. Carbon dioxide treatments were administered continuously while ozone treatments were applied 9∶00–16∶00 daily to better simulate natural exposure cycles. Experiments were repeated three times for a total of six replications (two replications per experiment); each replication was randomized in chambers and blocked by experiment (two block per experiment) in analysis to account for changes in environmental variables (light, temperature) across repeated experiments.

To capture pollen, polyethylene bags were placed over flowers upon emergence and held open. Bag and flower spike were removed following complete dehiscence of pollen and stored at −20°C until analysis.

Bagged flowers were washed three times with PBS-T (Phosphate Buffered Saline with 0.05% Tween 20 pH 7.4) to remove pollen; the amount of PBS-T was determined based on flower length and was measured precisely using a micro-pipette. Protein was extracted from the pollen by incubating at room temperature in the PBS-T solution for 2 hours. Pollen was separated by centrifugation at RCF 2,200×g. The extracted protein supernatant was stored at −20°C until allergen analysis. The pelleted pollen was suspended in a precisely measured volume of PBS-T solution and counted using a hemocytometer (3 times and averaged).

The Phl p 5 allergen content was determined using a monoclonal sandwich enzyme-linked immunosorbent assay (ELISA) (Indoor Biotechnologies, Inc., Charlottesville, VA, USA) using a mouse monoclonal IgG1 primary antibody to Phl p 5a&b, a 1% bovine serum albumin in PBS-T blocking agent, and a biotinolated mouse IgG1 secondary detection antibody to Phl p 5a&b. Concentrations were determined calorimetrically using streptavidin-peroxidase and substrate 1 mM ABTS (2,2′-azino-di-(3 ethylbenzthiazoline sulfonic acid)) in 70 mM citrate- phosphate buffer (pH 4.2). Serial dilutions of protein extracts were compared to a standard curve to quantify Phl p 5 allergen in each sample.

Analysis of variance of experimental data was used to account for treatment differences and was performed using a generalized linear model in SAS 9.3 (SAS Institute Inc, North Carolina, USA). Differences between treatment means were determined using Tukey- Kramer post-hoc test to account for uneven replications in response to treatments. Linear regressions were performed using analysis of covariance (ANCOVA) to evaluate relationship between co-factors.

## Results

**Figure 1:**
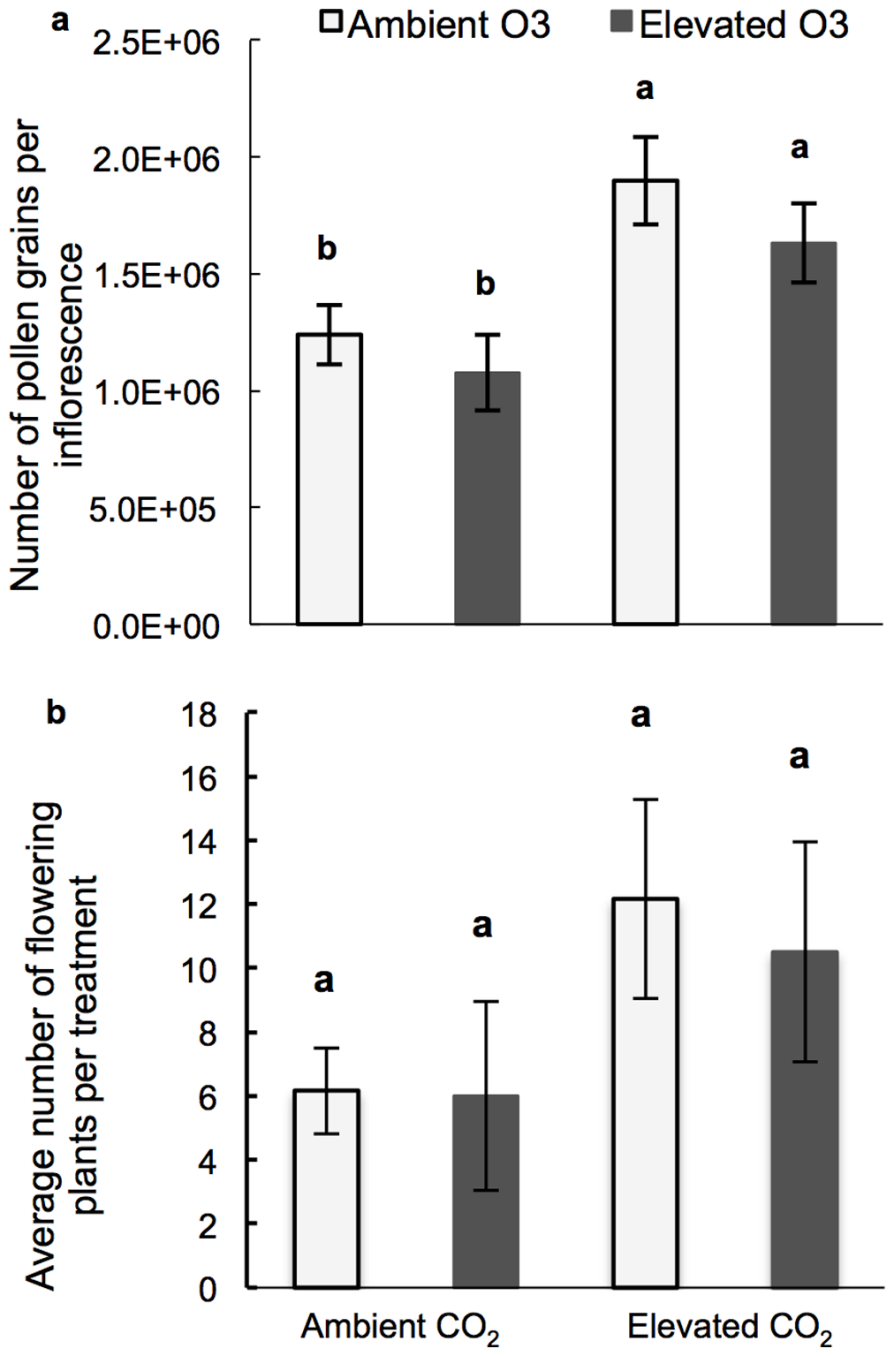
Effects on flowering and pollen production. **a**) Average pollen number per inflorescence. **b**) Average number of flowering plants per treatment. Ambient O_3_ = 30 ppb ozone; Elevated O_3_ = 80 ppb ozone; Ambient CO_2_ = 400 ppm carbon dioxide; Elevated CO_2_ = 800 ppm carbon dioxide. Significant differences (p<0.05) denoted by letters above bars determined by the Tukey-Kramer test.

Elevated CO_2_ (800 ppm) increased the amount of pollen produced by individual inflorescences by ∼53% whereas elevated O_3_ had no effect on the amount of pollen produced (Figure 1a; File S1). Since each plant only produces one inflorescence, the pollen output per plant was increased. The number of plants flowering per chamber was increased by elevated CO_2_, with and without elevated O_3_, during the experiment but was not statistically significant (Figure 1b).

**Figure 2:**
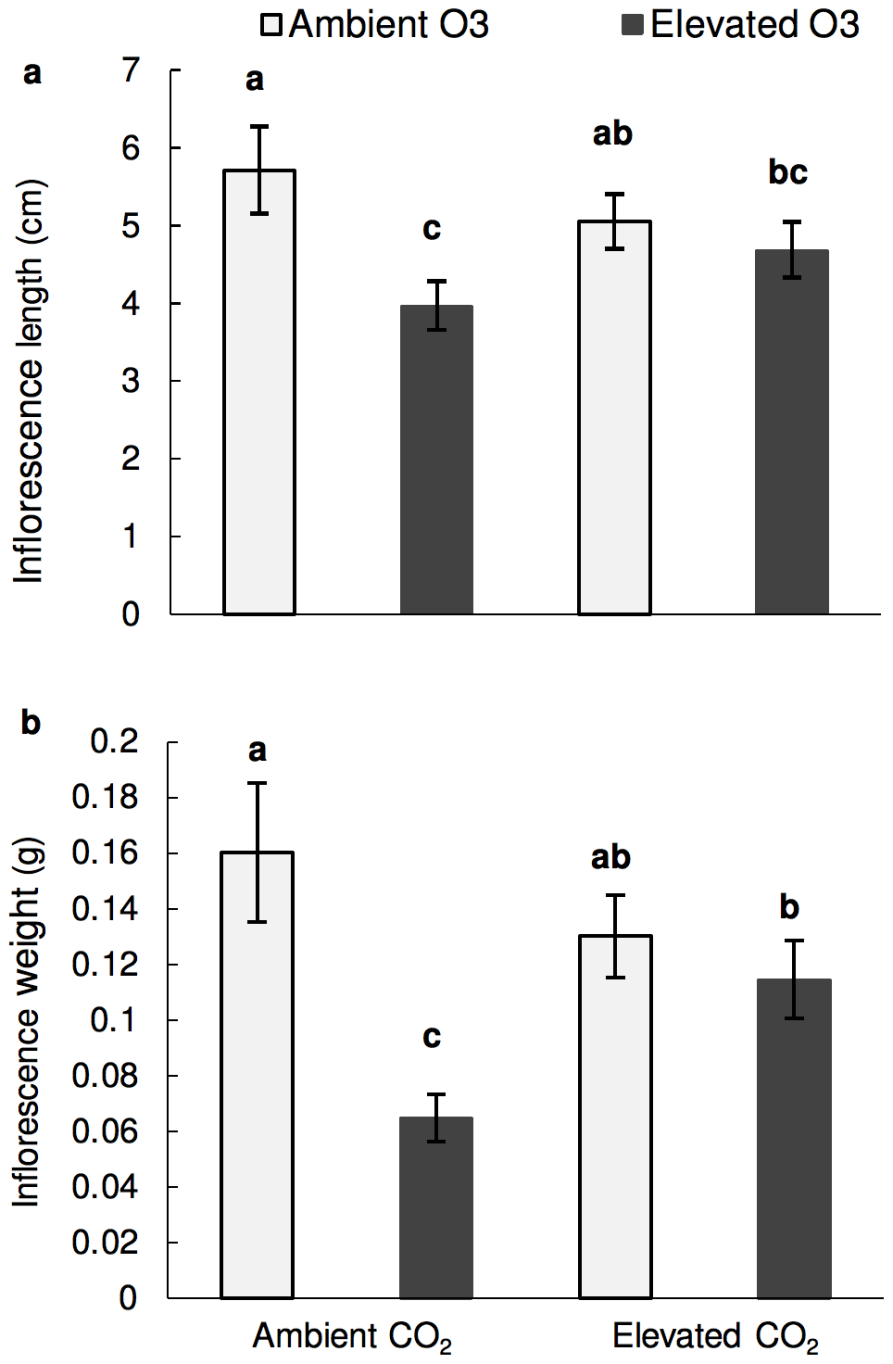
Effect on inflorescence size. **a**) inflorescence length (cm) **b**) Inflorescence weight (g) Ambient O_3_ = 30 ppb ozone; Elevated O_3_ = 80 ppb ozone; Ambient CO_2_ = 400 ppm carbon dioxide; Elevated CO_2_ = 800 ppm carbon dioxide. Significant differences (p<0.05) denoted by letters above bars were determined using the Tukey-Kramer test.

**Figure 3:**
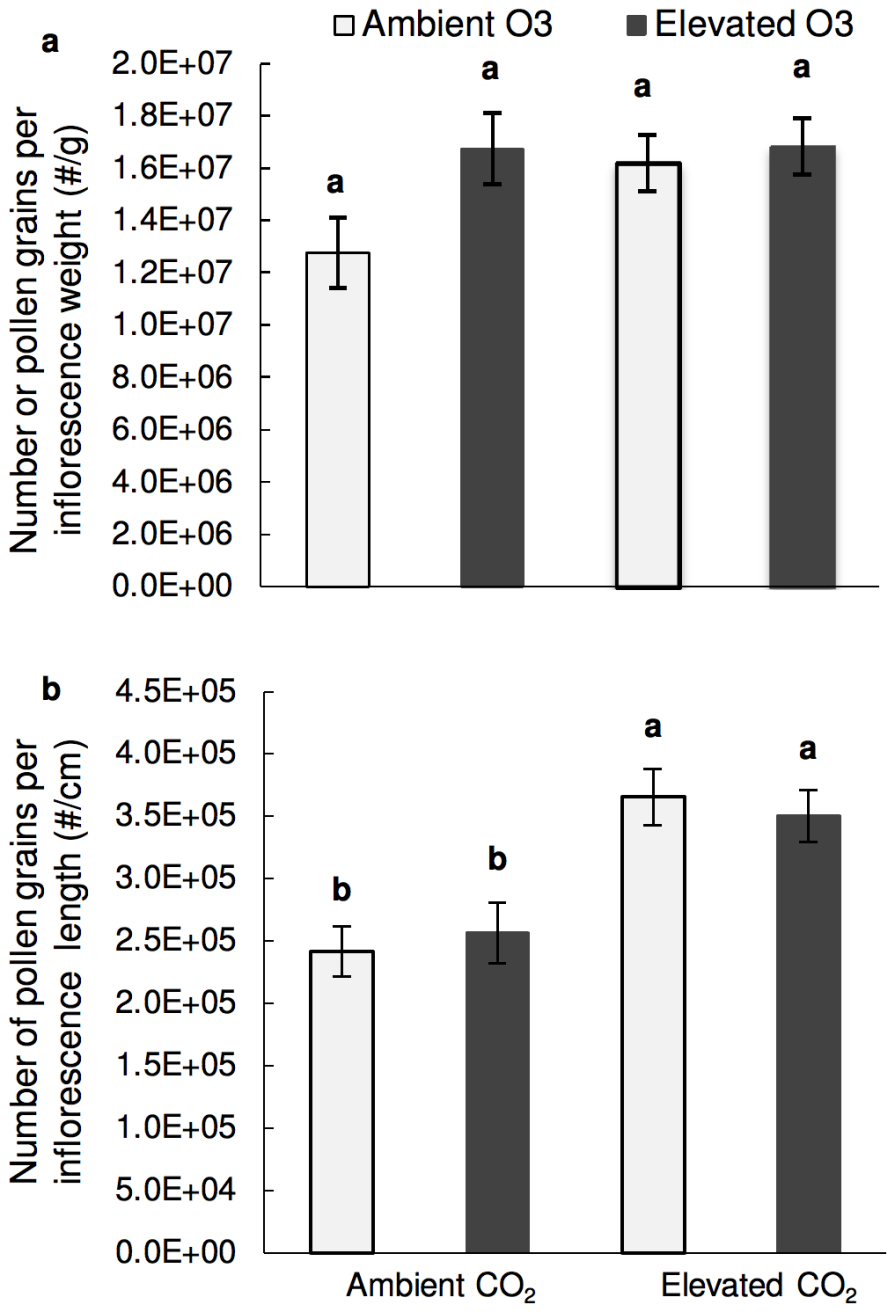
Relationship between pollen amount and inflorescence size. **a**) Pollen Number per length of inflorescence **b**) Pollen number per gram of inflorescence. Ambient O_3_ = 30 ppb ozone; Elevated O_3_ = 80 ppb ozone; Ambient CO_2_ = 400 ppm carbon dioxide; Elevated CO_2_ = 800 ppm carbon dioxide. Significant differences (p<0.05) denoted by letters above bars were determined using the Tukey-Kramer test.

Elevated CO_2_ did not stimulate an increase in inflorescence length or weight as has been found in ragweed (Figure 2a) [19]. However, our findings suggest that stimulation by CO_2_ offsets ozone damage and partially ameliorates O_3_–induced reductions in inflorescence size (Figure 2a, 2b). Furthermore, plants grown at elevated CO_2_ and O_3_ did not differ in the amount of pollen per weight (gram) of inflorescence than the control (Figure 3a). However, inflorescences of plants grown at elevated CO_2_ regardless of O_3_ level produced significantly more pollen per length of inflorescence (Figure 3b).

**Figure 4:**
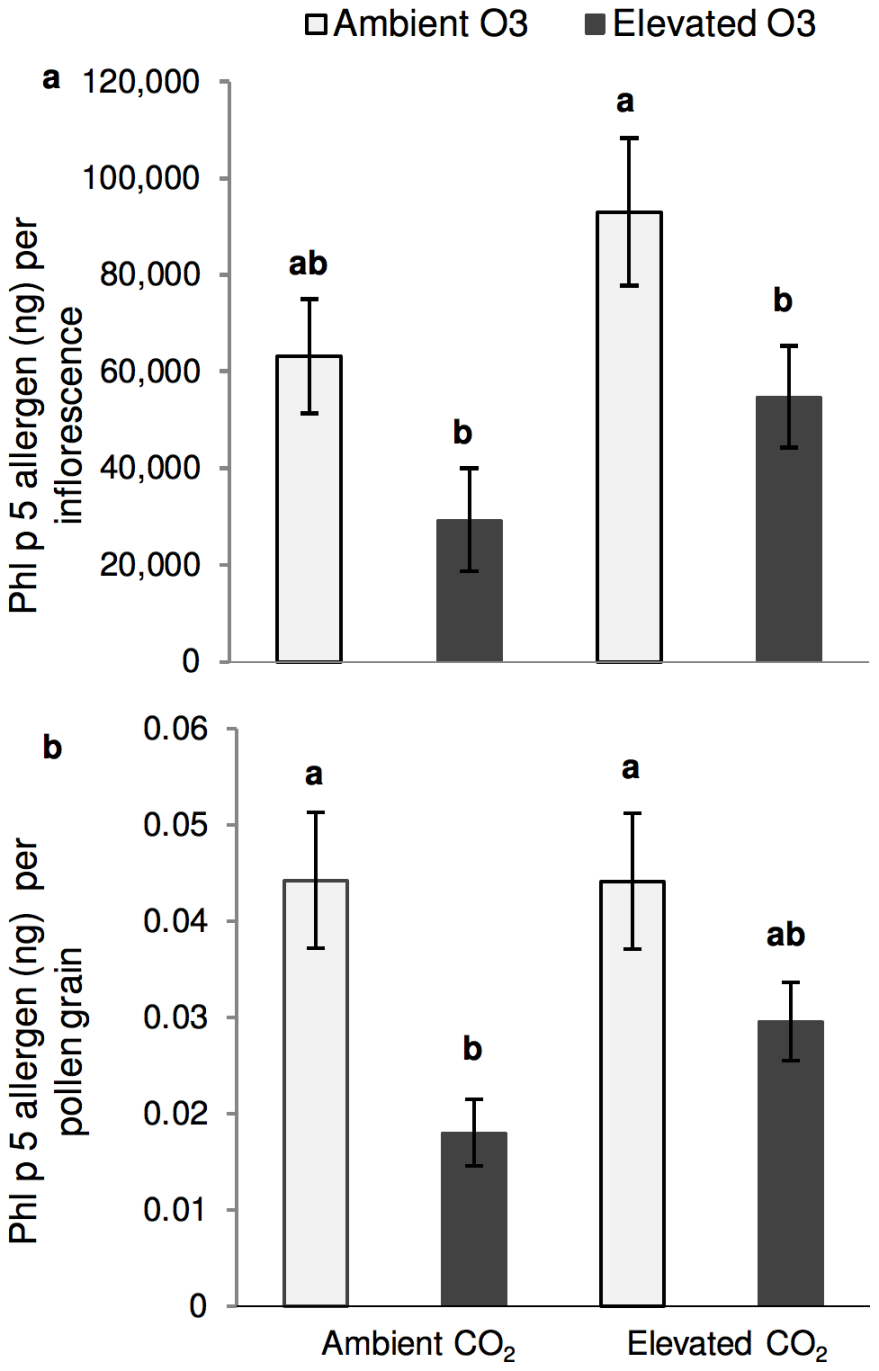
Phl p 5 allergen content. **a**) Average Phl p 5 concentration per inflorescence **b**) Average Phl p 5 concentration per pollen Ambient O_3_ = 30 ppb ozone; Elevated O_3_ = 80 ppb ozone; Ambient CO_2_ = 400 ppm carbon dioxide; Elevated CO_2_ = 800 ppm carbon dioxide. Significant differences (p<0.05) denoted by letters above bars determined by the Tukey-Kramer test.

Concentrations of Phl p 5 allergen per inflorescence and per pollen grain were not affected by CO_2_ levels but were reduced by elevated O_3._ (Figure 4a, 4b). Again, elevated CO_2_ partially ameliorated this reduction by elevated O_3_ (Figure 4b).

**Figure 5:**
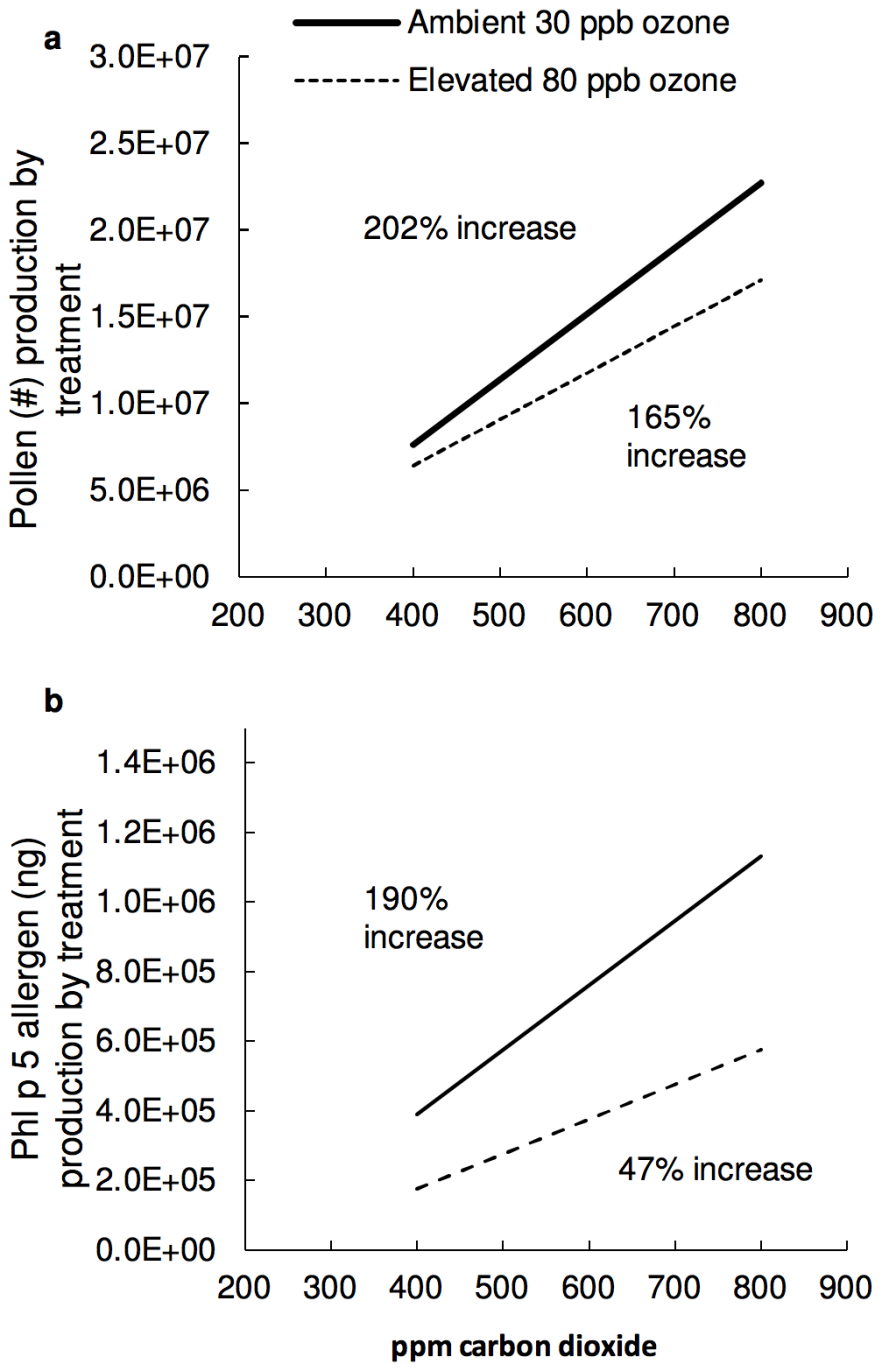
Predicted increases in pollen and allergen. **a**) Predicted increase in pollen production calculated by multiplying the average number of inflorescences in each treatment by the number of pollen produced per inflorescence. CO_2_ by O_3_ interaction is not significant indicating equal rates of increase with increasing CO_2_ **b**) Predicted increase in Phl p 5 allergen production calculated by multiplying the average number of inflorescences per treatment by the concentration of Phl p 5 per Inflorescence. The CO_2_ by O_3_ interaction was significant (ANCOVA p<0.05) indicating that elevated O_3_ reduced the rate of increase of Phl p 5 in response to elevated CO_2_. Solid line (−) determines trend at current ambient level of ozone (30 ppb). Dashed line (- - -) is future elevated level of ozone (80 ppb). Percentage number on graph indicates the percent increase in production with increasing CO_2_ levels.

Finally, to determine the overall changes in pollen and allergen production between current and projected CO_2_ and O_3_, we multiplied the average number of inflorescences produced per treatment by the number of pollen grains produced per inflorescence in that treatment (Figure 5a). Linear regression analysis determined a significant increase in pollen production of approximately 200% of current levels of CO_2_ to future predicted levels for both O_3_ treatments. The increase was only slightly affected by elevated O_3_ (202% at ambient O_3_, 165% at elevated O_3_), indicating that we can expect a large increase in pollen production due to increased CO_2_ regardless of future ozone levels.

Likewise, for Phl p 5 allergen levels, we multiplied the concentration of Phl p 5 per inflorescence by the average number of inflorescences per treatment. Linear regression analysis indicated a significant allergen increase at high CO_2_ concentrations with a larger 190% increase in Phl p 5 allergen production at ambient O_3_ than the 48% increase at elevated O_3_ (Figure 5b). Although the rate of increase of Phl p 5 concentration in response to elevated CO_2_ was slowed by elevated O_3_, the strong CO_2_-stimuation of pollen production suggests increased exposure to Timothy grass allergen overall.

## Discussion

We provide the first evidence that pollen production is significantly stimulated by elevated carbon dioxide in grasses, a finding that expands earlier work on the annual forb, common ragweed [9], [19] to another important allergenic plant taxonomic group. The inflorescences produced more pollen without increasing inflorescence size, indicating a greater number of male flowers per inflorescence and/or that male flowers in the inflorescence produced more pollen per anther.

The number of plants flowering per chamber was increased by elevated O_3_ or elevated CO_2_ during the experiment but was not statistically significant (Figure 1b). However, others have found an increased number of plants flowering in Timothy grass in response to elevated CO_2_, while elevated O_3_ had no impact on flowering [28]. In our study, the trend toward more plants flowering at elevated CO_2_ in Figure 1b indicates, however, that stimulation of flowering in Timothy grass warrants further research. Moreover, rising temperatures related to elevated CO_2_ may lengthen the flowering season thereby further increasing atmospheric pollen loads [19], [29], [30].

Biomass allocation in response to elevated carbon dioxide may be an important factor in pollen production [9], [20], [21]. In a concurrent study, biomass allocation was analyzed at different growth stages over the Timothy grass life cycle [31]. It was found that the highest level of biomass stimulation, particularly in the photosynthetic leaf tissue, occurred in the tillering stages leading up to the flowering stage, which could have contributed to increased energy available for pollen production during flowering.

In a previous study, ragweed increased Amb a 1 production, the major allergen in the pollen coat, in response to elevated levels of CO_2_ [10]. Ragweed differs in physiology from grasses; for instance, it is an annual plant, a dicot, and can be self-compatible, while grasses are perennial, monocots, and self-incompatible. The physiological importance of these two allergens may differ across these species [32]. Another explanation for lack of stimulation on Phl p 5 production could be that our work was conducted using moderate levels of nitrogen (∼5% N applied in solution once a week) and Phl p 5 protein production was potentially limited by nitrogen availability [17], [33].

Our experiment differed from other work on ozone in that we exposed our plants from seeding through dehiscence as would occur naturally with background O_3_ levels, while other experiments used more artificial exposures and durations [23]–[26]. In our study, the number of pollen grains produced per inflorescence was unaffected by O_3_; however, we did not test the viability of the pollen nor quantitate the maturity of the pollen, so we do not know the impacts on its function. There is a chance that our study underestimates the treatment effects on allergen concentration due to a change in recognition of the allergen by the monoclonal antibodies in our ELISA assay. Since ozone is a strong oxidant known to quickly degrade fatty acids and double bonds found in proteins [34], oxidative damage could have altered the Phl p 5 protein resulting in reduced recognition by the mouse IgG antibodies used in ELISA. Rogerieux et *al*. [26] came to similar conclusions, using IgE antibodies, when looking at multiple individual allergens found in Timothy grass. This degradation of allergen by ozone would also happen in the natural environment impacting recognition of allergens by human immune systems and hence is not exclusively an artifact of the experiment.

The implications of increasing CO_2_ for human health are clear. Stimulation of pollen production will increase airborne concentrations and increase exposure and suffering in pollen allergic individuals. The additional effects of increasing ozone are more complex. Ozone alone impacts human health negatively and has been shown to exacerbate the allergic airway response through irritation of the mucus membrane in respiratory pathways [35]. The projected O_3_ levels are nearing the current US National Ambient Air Quality standard (80 ppb pre-2008 and 75 ppb post-2008) used to protect human health [36]. Hence elevated levels would likely elicit negative respiratory health effects independent of any additional indirect health effects as a result of elevated CO_2_. The IPCC AR5 predicts lower O_3_ levels in some areas in the future due to air pollution mitigation strategies to reduce precursors to O_3_ and also climate feedbacks that affect O_3_ formation [37]. While increasing levels would increase airway irritation by ozone, lower levels of O_3_ would mean sustained levels of Phl p 5 allergen with higher Timothy grass pollen production resulting in increased allergen exposure and the potential for further worsening of allergic symptoms.

## Conclusion

Taken together, our results strongly suggest significant increases in grass pollen production and allergen exposure under future predicted levels of CO_2_ and O_3_ of 165–202%. Due to the widespread existence of grasses and their importance in eliciting allergic responses, these results indicate there will be a significant impact on human health worldwide as a result of future climate change.

## Supporting Information

File S1 **Data File.** Raw experimental data of flower number, flower weight, flower length, pollen count and ELISA Phl p 5 assay results used to produce this manuscript.
(PDF)
