# Supplementary material for: Projected Carbon Dioxide to Increase Grass Pollen and Allergen Exposure Despite Higher Ozone Levels
Source: PLoS One. 2014 Nov 5;9(11):e111712. doi: 10.1371/journal.pone.0111712 (PMC4221106; doi:10.1371/journal.pone.0111712)
Supplement: File S1 — Data File. Raw experimental data of flower number, flower weight, flower length, pollen count and ELISA Phl p 5 assay results used to produce this manuscript. (PDF) [file pone.0111712.s001.pdf]

experiment	block	trt	CO2	O3	Chamber	SUBREP	flower ID	CT 1	CT 2	CT3
FA11	2	4	2	2	1	1		NO FLOWERS PRODUCED IN THI		
FA11	1	2	1	2	2	1	1	147	153	
FA11	1	2	1	2	2	2	2	175	177	
FA11	1	2	1	2	2	3	3	194	167	
FA11	1	2	1	2	2	4	4	192	222	263
FA11	1	3	2	1	3	1	1	124	120	140
FA11	1	3	2	1	3	2	2	226	257	
FA11	1	3	2	1	3	3	3	192	172	
FA11	1	3	2	1	3	4	4			
FA11	1	3	2	1	3	5	5	FLOWERS DAMAGED NOT USED		
FA11	1	3	2	1	3	6	6	ANALYSIS OR PHL P 5 ANALYSIS		
FA11	1	3	2	1	3	7	7			
FA11	1	3	2	1	3	8	8	160	188	
FA11	1	3	2	1	3	9	9	166	151	185
FA11	1	3	2	1	3	10	10	119	153	
FA11	1	3	2	1	3	11	11	155	152	
FA11	1	3	2	1	3	12	12	109	112	
FA11	1	3	2	1	3	13	13	81	103	
FA11	1	3	2	1	3	14	14	206	174	
FA11	1	3	2	1	3	15	15	142	169	
FA11	1	1	1	1	4	1	1	142	151	
FA11	1	1	1	1	4	2	2	60	80	88
FA11	1	1	1	1	4	3	3	115	124	
FA11	1	1	1	1	4	4	4	91	89	
FA11	1	1	1	1	4	5	5	111	105	
FA11	1	1	1	1	4	6	6	121	127	
FA11	1	1	1	1	4	7	7	114	116	

experiment	block	trt	CO2	O3	Chamber	SUBREP	flower ID	CT 1	CT 2	CT3
FA11	1	1	1	1	4	8	8	99	98	
FA11	1	1	1	1	4	9	9	105	90	75
FA11	1	1	1	1	4	10	10	124	177	158
FA11	1	1	1	1	4	11	11	127	136	
FA11	1	4	2	2	5	1	1	228	193	208
FA11	1	4	2	2	5	2	2	237	228	
FA11	1	4	2	2	5	3	3	141	143	
FA11	1	4	2	2	5	4	4	63	64	
FA11	1	4	2	2	5	5	5	118	114	
FA11	1	4	2	2	5	6	6	76	73	
FA11	1	4	2	2	5	7	7	160	177	
FA11	1	4	2	2	5	8	8	229	238	
FA11	1	4	2	2	5	9	9	FLOWERS DAMAGED NOT USED		
FA11	1	4	2	2	5	10	10	195	122	181
FA11	1	4	2	2	5	11	11	133	142	
FA11	1	4	2	2	5	12	12	148	154	
FA11	1	4	2	2	5	13	13	171	152	
FA11	1	4	2	2	5	14	14	137	153	
FA11	1	4	2	2	5	15	15	FLOWERS DAMAGED NOT USED		
FA11	2	1	1	1	6	1	1	69	133	170
FA11	2	1	1	1	6	2	2	140	87	104
FA11	2	3	2	1	7	1	1	166	173	
FA11	2	3	2	1	7	2	2	221	215	281
FA11	2	3	2	1	7	3	3	165	172	
FA11	2	3	2	1	7	4	4	FLOWERS DAMAGED NOT USED		
FA11	2	3	2	1	7	5	5	222	272	229
FA11	2	3	2	1	7	6	6	FLOWERS DAMAGED NOT USED		

experiment	block	trt	CO2	O3	Chamber	SUBREP	flower ID	CT 1	CT 2	CT3
FA11	2	2	1	2	8	1		NO FLOWERS PRODUCED IN THI		
SP11	3	2	1	2	1	1	22	27	56	64
SP11	3	2	1	2	1	2	18A	39	32	33
SP11	3	2	1	2	1	3	18B	196	169	
SP11	3	2	1	2	1	4	24A	195	206	
SP11	3	2	1	2	1	5	24B	107	85	103
SP11	3	2	1	2	1	6	24C	232	214	
SP11	3	2	1	2	1	7	24D	249	276	
SP11	3	2	1	2	1	8	24E	42	37	51
SP11	3	2	1	2	1	9	29A	144	179	162
SP11	3	2	1	2	1	10	29B	32	41	33
SP11	3	2	1	2	1	11	32A	185	173	
SP11	3	2	1	2	1	12	32B	FLOWERS DAMAGED NOT USED		
SP11	3	2	1	2	1	13	32C	83	106	
SP11	3	4	2	2	2	1	36	198	163	172
SP11	3	4	2	2	2	2	40	94	94	
SP11	3	4	2	2	2	3	44	70	96	69
SP11	3	4	2	2	2	4	23A	273	296	
SP11	3	4	2	2	2	5	23B	119	105	
SP11	3	4	2	2	2	6	23C	111	98	
SP11	3	4	2	2	2	7	29A	138	118	
SP11	3	4	2	2	2	8	29B	148	133	
SP11	3	4	2	2	2	9	2A	96	81	
SP11	3	4	2	2	2	10	2B	308	314	297
SP11	3	4	2	2	2	11	2C	69	92	42
SP11	3	4	2	2	2	12	38A	224	227	
SP11	3	4	2	2	2	13	38B	131	121	

experiment	block	trt	CO2	O3	Chamber	SUBREP	flower ID	CT 1	CT 2	CT3
SP11	3	4	2	2	2	14	38C	103	88	89
SP11	3	4	2	2	2	15	43A	34	31	21
SP11	3	4	2	2	2	16	43B	FLOWERS DAMAGED NOT USED		
SP11	3	4	2	2	2	17	43C	167	211	
SP11	3	4	2	2	2	18	43D	237	218	
SP11	3	4	2	2	2	19	43E	94	109	118
SP11	3	4	2	2	2	20	45A	161	140	
SP11	3	4	2	2	2	21	45B	190	182	
SP11	3	4	2	2	2	22	45C	155	155	
SP11	3	1	1	1	3	1	6	91	80	
SP11	3	1	1	1	3	2	27	118	127	
SP11	3	1	1	1	3	3	37	FLOWERS DAMAGED NOT USED		
SP11	3	1	1	1	3	4	41	78	99	83
SP11	3	1	1	1	3	5	29A	116	128	130
SP11	3	1	1	1	3	6	29B	117	110	
SP11	3	1	1	1	3	7	29C	82	79	
SP11	3	1	1	1	3	8	9A	178	166	
SP11	3	1	1	1	3	9	9B	122	143	
SP11	3	3	2	1	4	1	16	FLOWERS DAMAGED NOT USED		
SP11	3	3	2	1	4	2	17	112	112	113
SP11	3	3	2	1	4	3	30	91	90	
SP11	3	3	2	1	4	4	31	FLOWERS DAMAGED NOT USED		
SP11	3	3	2	1	4	5	36	154	149	
SP11	3	3	2	1	4	6	20A	138	135	
SP11	3	3	2	1	4	7	20B	FLOWERS DAMAGED NOT USED		
SP11	3	3	2	1	4	8	24A	214	217	212
SP11	3	3	2	1	4	9	24B	9	10	15

experiment	block	trt	CO2	O3	Chamber	SUBREP	flower ID	CT 1	CT 2	CT3
SP11	3	3	2	1	4	10	24C	130	119	
SP11	3	3	2	1	4	11	25A	FLOWERS DAMAGED NOT USED		
SP11	3	3	2	1	4	12	25B	FLOWERS DAMAGED NOT USED		
SP11	3	3	2	1	4	13	25C	239	304	
SP11	3	3	2	1	4	14	27A	176	208	
SP11	3	3	2	1	4	15	27B	FLOWERS DAMAGED NOT USED		
SP11	3	3	2	1	4	16	27C	377	304	
SP11	3	3	2	1	4	17	33A	136	141	153
SP11	3	3	2	1	4	18	33B	333	295	
SP11	3	3	2	1	4	19	33C	170	202	
SP11	3	3	2	1	4	20	33D	199	151	
SP11	3	3	2	1	4	21	33E	FLOWERS DAMAGED NOT USED		
SP11	3	3	2	1	4	22	9A			
SP11	3	3	2	1	4	23	9B			
SP11	3	3	2	1	4	24	9C	130	137	
SP11	3	3	2	1	4	25	9D	289	313	
SP11	4	2	1	2	5	1	1	168	128	132
SP11	4	2	1	2	5	2	19	61	64	
SP11	4	2	1	2	5	3	20	18	23	21
SP11	4	2	1	2	5	4	22	FLOWERS DAMAGED NOT USED		
SP11	4	2	1	2	5	5	26	32	29	
SP11	4	2	1	2	5	6	38	84	108	119
SP11	4	2	1	2	5	7	41	315	302	
SP11	4	2	1	2	5	8	12A	86	128	132
SP11	4	2	1	2	5	9	12B	127	121	
SP11	4	2	1	2	5	10	14A	247	146	162
SP11	4	2	1	2	5	11	14B	214	145	

experiment	block	trt	CO2	O3	Chamber	SUBREP	flower ID	CT 1	CT 2	CT3
SP11	4	2	1	2	5	12	14C	110	180	
SP11	4	2	1	2	5	13	40A	115	91	101
SP11	4	2	1	2	5	14	40B	159	152	
SP11	4	2	1	2	5	15	40C	131	131	
SP11	4	2	1	2	5	16	40D	175	164	169
SP11	4	2	1	2	5	17	40E	45	35	31
SP11	4	3	2	1	6	1	5	141	169	
SP11	4	3	2	1	6	2	28	96	96	93
SP11	4	3	2	1	6	3	29	FLOWERS DAMAGED NOT USED		
SP11	4	3	2	1	6	4	37	59	70	78
SP11	4	3	2	1	6	5	25A	167	168	
SP11	4	3	2	1	6	6	25B	FLOWERS DAMAGED NOT USED		
SP11	4	3	2	1	6	7	27A	342	337	
SP11	4	3	2	1	6	8	27B	321	368	
SP11	4	3	2	1	6	9	27C	210	191	
SP11	4	3	2	1	6	10	27D	FLOWERS DAMAGED NOT USED		
SP11	4	3	2	1	6	11	27E			
SP11	4	3	2	1	6	12	42A			
SP11	4	3	2	1	6	13	42B	62	37	72
SP11	4	3	2	1	6	14	42C	FLOWERS DAMAGED NOT USED		
SP11	4	1	1	1	7	1	17	30	42	29
SP11	4	1	1	1	7	2	24	173	204	
SP11	4	1	1	1	7	3	27	FLOWERS DAMAGED NOT USED		
SP11	4	1	1	1	7	4	46	158	148	
SP11	4	1	1	1	7	5	9A	FLOWERS DAMAGED NOT USED		
SP11	4	1	1	1	7	6	9B			
SP11	4	4	2	2	8	1	6	263	253	

experiment	block	trt	CO2	O3	Chamber	SUBREP	flower ID	CT 1	CT 2	CT3
SP11	4	4	2	2	8	2	12	98	131	
SP11	4	4	2	2	8	3	28	89	135	102
SP11	4	4	2	2	8	4	42	306	336	328
SP11	4	4	2	2	8	5	45	139	206	187
SP11	4	4	2	2	8	6	19A	FLOWERS DAMAGED NOT USED		
SP11	4	4	2	2	8	7	19B	49	66	54
SP11	4	4	2	2	8	8	19C	50	84	88
SP11	4	4	2	2	8	9	29A	23	36	41
SP11	4	4	2	2	8	10	29B	158	178	
SP11	4	4	2	2	8	11	39A	FLOWERS DAMAGED NOT USED		
SP11	4	4	2	2	8	12	39B	79	106	67
SP11	4	4	2	2	8	13	3A	364	298	
SP11	4	4	2	2	8	14	3B	95	82	
SP11	4	4	2	2	8	15	3C	123	153	
SP11	4	4	2	2	8	16	3D	320	280	
SP12	5	4	2	2	1	1	1	126	124	
SP12	5	4	2	2	1	2	2	174	193	153
SP12	5	4	2	2	1	3	3	282	279	239
SP12	5	4	2	2	1	4	4	235	234	
SP12	5	4	2	2	1	5	5	270	255	
SP12	5	1	1	1	2	1	1	195	129	216
SP12	5	1	1	1	2	2	2	183	190	
SP12	5	1	1	1	2	3	3	103	93	
SP12	5	1	1	1	2	4	4	92	84	
SP12	5	1	1	1	2	5	5	56	63	
SP12	5	3	2	1	3	1	1	122	102	129
SP12	5	3	2	1	3	2	2	110	98	76

experiment	block	trt	CO2	O3	Chamber	SUBREP	flower ID	CT 1	CT 2	CT3
SP12	5	3	2	1	3	3	3	154	84	135
SP12	5	3	2	1	3	4	4	290	242	248
SP12	5	3	2	1	3	5	5	107	119	
SP12	5	3	2	1	3	6	6	FLOWERS DAMAGED NOT USED		
SP12	5	3	2	1	3	7	7	193	184	147
SP12	5	3	2	1	3	8	8	FLOWERS DAMAGED NOT USED		
SP12	5	3	2	1	3	9	9	148	206	154
SP12	6	1	1	1	4	1	1	204	205	
SP12	6	1	1	1	4	2	2	172	132	99
SP12	6	1	1	1	4	3	3	209	293	247
SP12	6	1	1	1	4	4	4	158	166	
SP12	6	4	2	2	5	1	1	189	169	215
SP12	6	4	2	2	5	2	2	188	215	158
SP12	6	4	2	2	5	3	3	200	154	131
SP12	6	4	2	2	5	4	4	184	181	
SP12	6	4	2	2	5	5	5	138	139	
SP12	5	2	1	2	6	1		NO FLOWERS PRODUCED IN THI		
SP12	6	3	2	1	7	1	1	FLOWERS DAMAGED NOT USED		
SP12	6	3	2	1	7	2	2			
SP12	6	3	2	1	7	3	3	51	50	39
SP12	6	3	2	1	7	4	4	238	253	
SP12	5	2	1	2	8	1	1	62	58	
SP12	5	2	1	2	8	2	2	51	41	

Phleum pratense L.

treatments

experiment	block	trt	CO2	O3	Chamber	SUBREP	flower ID	CT 1	CT 2	CT3
	ambient (1)	elevated (2)			trt 1	trt 2	trt 3	trt 4		
CO2	400 ppm	800 ppm			30 ppb O3	80 ppb O3	30 ppb O3	80 ppb O3		
O3	30 ppb	80 ppb			400 ppm CO2	400 ppm C	800 ppm C	800 ppm CO2		
3 experiment	FA11	SP11	SP12							
8 chambers per experiment in a complete factorial design										
2 reps (block) per experiment										
Chamber experiment (CSTR)										
All flowers collected from each chamber										
flower bagged upon emergence and immediately removed and placed in freezer after dehiscence of pollen										
Flowers washed with PBS-T (3 washes; pollen removed from each wash via centrifuge; three washes combined for protein analysis)										
pollen suspended in known volume and counted using hemocytometer (the total amount of pollen from each wash counted separately										
Phl-p-5 content determined using ELISA										
ALL FLOWERS WERE COLLECTED FROM ALL CHAMBERS										
Some flowers were damaged in collection (ie pollen lost) and not used in analysis of pollen content or phl p 5 analysis but flower counte										

								WASH 1		
experiment	block	trt	CO2	O3	Chamber	SUBREP	flower ID	CT4	AVERAGE	WASH UL
FA11	2	4	2	2	1	1		S CHAMBER		
FA11	1	2	1	2	2	1	1		150	1000
FA11	1	2	1	2	2	2	2		176	1500
FA11	1	2	1	2	2	3	3		180.5	1500
FA11	1	2	1	2	2	4	4	90	191.75	1000
FA11	1	3	2	1	3	1	1	107	122.75	1000
FA11	1	3	2	1	3	2	2		241.5	1500
FA11	1	3	2	1	3	3	3		182	1500
FA11	1	3	2	1	3	4	4			
FA11	1	3	2	1	3	5	5	IN POLLEN		
FA11	1	3	2	1	3	6	6			
FA11	1	3	2	1	3	7	7			
FA11	1	3	2	1	3	8	8		174	1500
FA11	1	3	2	1	3	9	9	131	158.25	1000
FA11	1	3	2	1	3	10	10		136	1500
FA11	1	3	2	1	3	11	11		153.5	500
FA11	1	3	2	1	3	12	12		110.5	1500
FA11	1	3	2	1	3	13	13		92	1500
FA11	1	3	2	1	3	14	14		190	1500
FA11	1	3	2	1	3	15	15		155.5	1500
FA11	1	1	1	1	4	1	1		146.5	500
FA11	1	1	1	1	4	2	2	85	78.25	1000
FA11	1	1	1	1	4	3	3		119.5	1000
FA11	1	1	1	1	4	4	4		90	1000
FA11	1	1	1	1	4	5	5		108	500
FA11	1	1	1	1	4	6	6		124	1000
FA11	1	1	1	1	4	7	7		115	500

								WASH 1		
experiment	block	trt	CO2	O3	Chamber	SUBREP	flower ID	CT4	AVERAGE	WASH UL
FA11	1	1	1	1	4	8	8		98.5	1500
FA11	1	1	1	1	4	9	9	82	88	1000
FA11	1	1	1	1	4	10	10	168	156.75	1500
FA11	1	1	1	1	4	11	11		131.5	500
FA11	1	4	2	2	5	1	1	198	206.75	1500
FA11	1	4	2	2	5	2	2		232.5	1500
FA11	1	4	2	2	5	3	3		142	1000
FA11	1	4	2	2	5	4	4		63.5	1000
FA11	1	4	2	2	5	5	5		116	1500
FA11	1	4	2	2	5	6	6		74.5	1000
FA11	1	4	2	2	5	7	7		168.5	1500
FA11	1	4	2	2	5	8	8		233.5	1500
FA11	1	4	2	2	5	9	9	IN POLLEN/PHL P 5 ANALYSIS		
FA11	1	4	2	2	5	10	10	153	162.75	1500
FA11	1	4	2	2	5	11	11		137.5	1500
FA11	1	4	2	2	5	12	12		151	1500
FA11	1	4	2	2	5	13	13		161.5	1000
FA11	1	4	2	2	5	14	14		145	1500
FA11	1	4	2	2	5	15	15	IN POLLEN/PHL P 5 ANALYSIS		
FA11	2	1	1	1	6	1	1	102	118.5	1000
FA11	2	1	1	1	6	2	2	81	103	1000
FA11	2	3	2	1	7	1	1		169.5	1500
FA11	2	3	2	1	7	2	2		239	500
FA11	2	3	2	1	7	3	3		168.5	1500
FA11	2	3	2	1	7	4	4	IN POLLEN/PHL P 5 ANALYSIS		
FA11	2	3	2	1	7	5	5	241	241	1500
FA11	2	3	2	1	7	6	6	IN POLLEN/PHL P 5 ANALYSIS		

								WASH 1		
experiment	block	trt	CO2	O3	Chamber	SUBREP	flower ID	CT4	AVERAGE	WASH UL
FA11	2	2	1	2	8	1		S CHAMBER		
SP11	3	2	1	2	1	1	22		49	500
SP11	3	2	1	2	1	2	18A		34.66667	500
SP11	3	2	1	2	1	3	18B		182.5	500
SP11	3	2	1	2	1	4	24A		200.5	500
SP11	3	2	1	2	1	5	24B	106	100.25	500
SP11	3	2	1	2	1	6	24C		223	500
SP11	3	2	1	2	1	7	24D		262.5	500
SP11	3	2	1	2	1	8	24E		43.33333	500
SP11	3	2	1	2	1	9	29A		161.6667	500
SP11	3	2	1	2	1	10	29B		35.33333	500
SP11	3	2	1	2	1	11	32A		179	500
SP11	3	2	1	2	1	12	32B	IN POLLEN/PHL P 5 ANALYSIS		
SP11	3	2	1	2	1	13	32C		94.5	500
SP11	3	4	2	2	2	1	36		177.6667	500
SP11	3	4	2	2	2	2	40		94	500
SP11	3	4	2	2	2	3	44		78.33333	500
SP11	3	4	2	2	2	4	23A		284.5	500
SP11	3	4	2	2	2	5	23B		112	500
SP11	3	4	2	2	2	6	23C		104.5	500
SP11	3	4	2	2	2	7	29A		128	500
SP11	3	4	2	2	2	8	29B		140.5	500
SP11	3	4	2	2	2	9	2A		88.5	500
SP11	3	4	2	2	2	10	2B		306.3333	500
SP11	3	4	2	2	2	11	2C	74	69.25	500
SP11	3	4	2	2	2	12	38A		225.5	500
SP11	3	4	2	2	2	13	38B		126	500

								WASH 1		
experiment	block	trt	CO2	O3	Chamber	SUBREP	flower ID	CT4	AVERAGE	WASH UL
SP11	3	4	2	2	2	14	38C		93.33333	500
SP11	3	4	2	2	2	15	43A		28.66667	500
SP11	3	4	2	2	2	16	43B	IN POLLEN/PHL P 5 ANALYSIS		
SP11	3	4	2	2	2	17	43C		189	500
SP11	3	4	2	2	2	18	43D		227.5	500
SP11	3	4	2	2	2	19	43E		107	500
SP11	3	4	2	2	2	20	45A		150.5	500
SP11	3	4	2	2	2	21	45B		186	500
SP11	3	4	2	2	2	22	45C		155	500
SP11	3	1	1	1	3	1	6		85.5	500
SP11	3	1	1	1	3	2	27		122.5	500
SP11	3	1	1	1	3	3	37	IN POLLEN/PHL P 5 ANALYSIS		
SP11	3	1	1	1	3	4	41		86.66667	500
SP11	3	1	1	1	3	5	29A		124.6667	500
SP11	3	1	1	1	3	6	29B		113.5	500
SP11	3	1	1	1	3	7	29C		80.5	500
SP11	3	1	1	1	3	8	9A		172	500
SP11	3	1	1	1	3	9	9B		132.5	500
SP11	3	3	2	1	4	1	16	IN POLLEN/PHL P 5 ANALYSIS		
SP11	3	3	2	1	4	2	17	98	108.75	500
SP11	3	3	2	1	4	3	30		90.5	500
SP11	3	3	2	1	4	4	31	IN POLLEN/PHL P 5 ANALYSIS		
SP11	3	3	2	1	4	5	36		151.5	500
SP11	3	3	2	1	4	6	20A		136.5	500
SP11	3	3	2	1	4	7	20B	IN POLLEN/PHL P 5 ANALYSIS		
SP11	3	3	2	1	4	8	24A		214.3333	500
SP11	3	3	2	1	4	9	24B		11.33333	500

								WASH 1		
experiment	block	trt	CO2	O3	Chamber	SUBREP	flower ID	CT4	AVERAGE	WASH UL
SP11	3	3	2	1	4	10	24C		124.5	500
SP11	3	3	2	1	4	11	25A	IN POLLEN/PHL P 5 ANALYSIS		
SP11	3	3	2	1	4	12	25B	IN POLLEN/PHL P 5 ANALYSIS		
SP11	3	3	2	1	4	13	25C		271.5	500
SP11	3	3	2	1	4	14	27A		192	500
SP11	3	3	2	1	4	15	27B	IN POLLEN/PHL P 5 ANALYSIS		
SP11	3	3	2	1	4	16	27C		340.5	500
SP11	3	3	2	1	4	17	33A		143.3333	500
SP11	3	3	2	1	4	18	33B		314	500
SP11	3	3	2	1	4	19	33C		186	500
SP11	3	3	2	1	4	20	33D		175	500
SP11	3	3	2	1	4	21	33E	IN POLLEN/PHL P 5 ANALYSIS		
SP11	3	3	2	1	4	22	9A			
SP11	3	3	2	1	4	23	9B			
SP11	3	3	2	1	4	24	9C		133.5	500
SP11	3	3	2	1	4	25	9D		301	500
SP11	4	2	1	2	5	1	1	126	138.5	500
SP11	4	2	1	2	5	2	19		62.5	500
SP11	4	2	1	2	5	3	20		20.66667	500
SP11	4	2	1	2	5	4	22	IN POLLEN/PHL P 5 ANALYSIS		
SP11	4	2	1	2	5	5	26		30.5	500
SP11	4	2	1	2	5	6	38		103.6667	500
SP11	4	2	1	2	5	7	41		308.5	500
SP11	4	2	1	2	5	8	12A		115.3333	500
SP11	4	2	1	2	5	9	12B		124	500
SP11	4	2	1	2	5	10	14A		185	500
SP11	4	2	1	2	5	11	14B		179.5	500

								WASH 1		
experiment	block	trt	CO2	O3	Chamber	SUBREP	flower ID	CT4	AVERAGE	WASH UL
SP11	4	2	1	2	5	12	14C		145	500
SP11	4	2	1	2	5	13	40A		102.3333	500
SP11	4	2	1	2	5	14	40B		155.5	500
SP11	4	2	1	2	5	15	40C		131	500
SP11	4	2	1	2	5	16	40D	164	168	500
SP11	4	2	1	2	5	17	40E		37	500
SP11	4	3	2	1	6	1	5		155	500
SP11	4	3	2	1	6	2	28		95	500
SP11	4	3	2	1	6	3	29	IN POLLEN/PHL P 5 ANALYSIS		
SP11	4	3	2	1	6	4	37		69	500
SP11	4	3	2	1	6	5	25A		167.5	500
SP11	4	3	2	1	6	6	25B	IN POLLEN/PHL P 5 ANALYSIS		
SP11	4	3	2	1	6	7	27A		339.5	500
SP11	4	3	2	1	6	8	27B		344.5	500
SP11	4	3	2	1	6	9	27C		200.5	500
SP11	4	3	2	1	6	10	27D	IN POLLEN/PHL P 5 ANALYSIS		
SP11	4	3	2	1	6	11	27E			
SP11	4	3	2	1	6	12	42A			
SP11	4	3	2	1	6	13	42B	58	57.25	500
SP11	4	3	2	1	6	14	42C	IN POLLEN/PHL P 5 ANALYSIS		
SP11	4	1	1	1	7	1	17		33.66667	500
SP11	4	1	1	1	7	2	24		188.5	500
SP11	4	1	1	1	7	3	27	IN POLLEN/PHL P 5 ANALYSIS		
SP11	4	1	1	1	7	4	46		153	500
SP11	4	1	1	1	7	5	9A	IN POLLEN/PHL P 5 ANALYSIS		
SP11	4	1	1	1	7	6	9B			
SP11	4	4	2	2	8	1	6		258	500

								WASH 1		
experiment	block	trt	CO2	O3	Chamber	SUBREP	flower ID	CT4	AVERAGE	WASH UL
SP11	4	4	2	2	8	2	12		114.5	500
SP11	4	4	2	2	8	3	28	126	113	500
SP11	4	4	2	2	8	4	42		323.3333	500
SP11	4	4	2	2	8	5	45	194	181.5	500
SP11	4	4	2	2	8	6	19A	IN POLLEN/PHL P 5 ANALYSIS		
SP11	4	4	2	2	8	7	19B		56.33333	500
SP11	4	4	2	2	8	8	19C		74	500
SP11	4	4	2	2	8	9	29A		33.33333	500
SP11	4	4	2	2	8	10	29B		168	500
SP11	4	4	2	2	8	11	39A	IN POLLEN/PHL P 5 ANALYSIS		
SP11	4	4	2	2	8	12	39B		84	500
SP11	4	4	2	2	8	13	3A		331	500
SP11	4	4	2	2	8	14	3B		88.5	500
SP11	4	4	2	2	8	15	3C		138	500
SP11	4	4	2	2	8	16	3D		300	500
SP12	5	4	2	2	1	1	1		125	500
SP12	5	4	2	2	1	2	2	160	170	500
SP12	5	4	2	2	1	3	3	307	276.75	500
SP12	5	4	2	2	1	4	4		234.5	500
SP12	5	4	2	2	1	5	5		262.5	500
SP12	5	1	1	1	2	1	1	217	189.25	500
SP12	5	1	1	1	2	2	2		186.5	500
SP12	5	1	1	1	2	3	3		98	500
SP12	5	1	1	1	2	4	4		88	500
SP12	5	1	1	1	2	5	5		59.5	500
SP12	5	3	2	1	3	1	1	102	113.75	1500
SP12	5	3	2	1	3	2	2	94	94.5	500

								WASH 1		
experiment	block	trt	CO2	O3	Chamber	SUBREP	flower ID	CT4	AVERAGE	WASH UL
SP12	5	3	2	1	3	3	3	123	124	500
SP12	5	3	2	1	3	4	4	210	247.5	500
SP12	5	3	2	1	3	5	5		113	500
SP12	5	3	2	1	3	6	6	IN POLLEN/PHL P 5 ANALYSIS		
SP12	5	3	2	1	3	7	7	216	185	500
SP12	5	3	2	1	3	8	8	IN POLLEN/PHL P 5 ANALYSIS		
SP12	5	3	2	1	3	9	9	149	164.25	1000
SP12	6	1	1	1	4	1	1		204.5	1000
SP12	6	1	1	1	4	2	2	106	127.25	500
SP12	6	1	1	1	4	3	3	209	239.5	500
SP12	6	1	1	1	4	4	4		162	500
SP12	6	4	2	2	5	1	1	246	204.75	500
SP12	6	4	2	2	5	2	2	237	199.5	1000
SP12	6	4	2	2	5	3	3	163	162	1000
SP12	6	4	2	2	5	4	4		182.5	500
SP12	6	4	2	2	5	5	5		138.5	1000
SP12	5	2	1	2	6	1		S CHAMBER		
SP12	6	3	2	1	7	1	1	IN POLLEN/PHL P 5 ANALYSIS		
SP12	6	3	2	1	7	2	2			
SP12	6	3	2	1	7	3	3	62	50.5	500
SP12	6	3	2	1	7	4	4		245.5	1000
SP12	5	2	1	2	8	1	1		60	500
SP12	5	2	1	2	8	2	2		46	500
Phleum pratense L.										
treatments										

								WASH 1		
experiment	block	trt	CO2	O3	Chamber	SUBREP	flower ID	CT4	AVERAGE	WASH UL
	ambient (1)	elevated (2)			trt 1	trt 2	trt 3			
CO2	400 ppm	800 ppm			30 ppb O3	80 ppb O3	30 ppb O3			
O3	30 ppb	80 ppb			400 ppm CO2	400 ppm C	800 ppm C			
3 experiment	FA11	SP11	SP12							
8 chambers per experiment in a complete factorial design										
2 reps (block) per experiment										
Chamber experiment (CSTR)										
All flowers collected from each chamber										
flower bagged upon emergence and immediately removed and placed in freezer after dehiscence of pc										
Flowers washed with PBS-T (3 washes; pollen removed from each wash via centrifuge; three washes cc										
pollen suspended in known volume and counted using hemocytometer (the total amount of pollen fro								to determine wash efficiency)		
Phl-p-5 content determined using ELISA										
ALL FLOWERS WERE COLLECTED FROM ALL CHAMBERS										
Some flowers were damaged in collection (ie pollen lost) and not used in analysis of pollen content or p								d as a flower produced in treatm		

experiment	block	trt	CO2	O3	Chamber	SUBREP	flower ID	TTL POL	CT1	CT2
FA11	2	4	2	2	1	1				
FA11	1	2	1	2	2	1	1	1500000	61	58
FA11	1	2	1	2	2	2	2	2640000	70	80
FA11	1	2	1	2	2	3	3	2707500	94	89
FA11	1	2	1	2	2	4	4	1917500	84	98
FA11	1	3	2	1	3	1	1	1227500	35	59
FA11	1	3	2	1	3	2	2	3622500	74	68
FA11	1	3	2	1	3	3	3	2730000	84	85
FA11	1	3	2	1	3	4	4			
FA11	1	3	2	1	3	5	5			
FA11	1	3	2	1	3	6	6			
FA11	1	3	2	1	3	7	7			
FA11	1	3	2	1	3	8	8	2610000	101	88
FA11	1	3	2	1	3	9	9	1582500	93	88
FA11	1	3	2	1	3	10	10	2040000	86	91
FA11	1	3	2	1	3	11	11	767500	47	44
FA11	1	3	2	1	3	12	12	1657500	77	71
FA11	1	3	2	1	3	13	13	1380000	68	54
FA11	1	3	2	1	3	14	14	2850000	90	107
FA11	1	3	2	1	3	15	15	2332500	66	66
FA11	1	1	1	1	4	1	1	732500	54	43
FA11	1	1	1	1	4	2	2	782500	42	72
FA11	1	1	1	1	4	3	3	1195000	66	62
FA11	1	1	1	1	4	4	4	900000	90	98
FA11	1	1	1	1	4	5	5	540000	50	61
FA11	1	1	1	1	4	6	6	1240000	90	88
FA11	1	1	1	1	4	7	7	575000	52	44

experiment	block	trt	CO2	O3	Chamber	SUBREP	flower ID	TTL POL	CT1	CT2
FA11	1	1	1	1	4	8	8	1477500	86	82
FA11	1	1	1	1	4	9	9	880000	22	44
FA11	1	1	1	1	4	10	10	2351250	88	112
FA11	1	1	1	1	4	11	11	657500	105	96
FA11	1	4	2	2	5	1	1	3101250	100	88
FA11	1	4	2	2	5	2	2	3487500	120	110
FA11	1	4	2	2	5	3	3	1420000	85	66
FA11	1	4	2	2	5	4	4	635000	35	43
FA11	1	4	2	2	5	5	5	1740000	72	71
FA11	1	4	2	2	5	6	6	745000	52	63
FA11	1	4	2	2	5	7	7	2527500	149	145
FA11	1	4	2	2	5	8	8	3502500	148	142
FA11	1	4	2	2	5	9	9			
FA11	1	4	2	2	5	10	10	2441250	93	86
FA11	1	4	2	2	5	11	11	2062500	43	43
FA11	1	4	2	2	5	12	12	2265000	139	161
FA11	1	4	2	2	5	13	13	1615000	54	48
FA11	1	4	2	2	5	14	14	2175000	122	104
FA11	1	4	2	2	5	15	15			
FA11	2	1	1	1	6	1	1	1185000	79	71
FA11	2	1	1	1	6	2	2	1030000	58	61
FA11	2	3	2	1	7	1	1	2542500	123	82
FA11	2	3	2	1	7	2	2	1195000	60	65
FA11	2	3	2	1	7	3	3	2527500	92	70
FA11	2	3	2	1	7	4	4			
FA11	2	3	2	1	7	5	5	3615000	92	91
FA11	2	3	2	1	7	6	6			

experiment	block	trt	CO2	O3	Chamber	SUBREP	flower ID	TTL POL	CT1	CT2
FA11	2	2	1	2	8	1				
SP11	3	2	1	2	1	1	22	245000	2	4
SP11	3	2	1	2	1	2	18A	173333.3	30	5
SP11	3	2	1	2	1	3	18B	912500	47	36
SP11	3	2	1	2	1	4	24A	1002500	37	45
SP11	3	2	1	2	1	5	24B	501250	31	36
SP11	3	2	1	2	1	6	24C	1115000	28	35
SP11	3	2	1	2	1	7	24D	1312500	38	43
SP11	3	2	1	2	1	8	24E	216666.7	12	13
SP11	3	2	1	2	1	9	29A	808333.3	23	23
SP11	3	2	1	2	1	10	29B	176666.7	8	6
SP11	3	2	1	2	1	11	32A	895000	34	34
SP11	3	2	1	2	1	12	32B			
SP11	3	2	1	2	1	13	32C	472500	10	18
SP11	3	4	2	2	2	1	36	888333.3	71	68
SP11	3	4	2	2	2	2	40	470000	17	31
SP11	3	4	2	2	2	3	44	391666.7	11	13
SP11	3	4	2	2	2	4	23A	1422500	83	62
SP11	3	4	2	2	2	5	23B	560000	23	22
SP11	3	4	2	2	2	6	23C	522500	57	44
SP11	3	4	2	2	2	7	29A	640000	20	22
SP11	3	4	2	2	2	8	29B	702500	33	17
SP11	3	4	2	2	2	9	2A	442500	17	24
SP11	3	4	2	2	2	10	2B	1531667	91	58
SP11	3	4	2	2	2	11	2C	346250	16	25
SP11	3	4	2	2	2	12	38A	1127500	38	33
SP11	3	4	2	2	2	13	38B	630000	20	18

experiment	block	trt	CO2	O3	Chamber	SUBREP	flower ID	TTL POL	CT1	CT2
SP11	3	4	2	2	2	14	38C	466666.7	9	11
SP11	3	4	2	2	2	15	43A	143333.3	15	10
SP11	3	4	2	2	2	16	43B			
SP11	3	4	2	2	2	17	43C	945000	92	94
SP11	3	4	2	2	2	18	43D	1137500	44	48
SP11	3	4	2	2	2	19	43E	535000	41	30
SP11	3	4	2	2	2	20	45A	752500	18	24
SP11	3	4	2	2	2	21	45B	930000	46	60
SP11	3	4	2	2	2	22	45C	775000	41	33
SP11	3	1	1	1	3	1	6	427500	11	13
SP11	3	1	1	1	3	2	27	612500	15	23
SP11	3	1	1	1	3	3	37			
SP11	3	1	1	1	3	4	41	433333.3	6	7
SP11	3	1	1	1	3	5	29A	623333.3	18	13
SP11	3	1	1	1	3	6	29B	567500	37	36
SP11	3	1	1	1	3	7	29C	402500	18	16
SP11	3	1	1	1	3	8	9A	860000	16	23
SP11	3	1	1	1	3	9	9B	662500	30	27
SP11	3	3	2	1	4	1	16			
SP11	3	3	2	1	4	2	17	543750	63	76
SP11	3	3	2	1	4	3	30	452500	20	27
SP11	3	3	2	1	4	4	31			
SP11	3	3	2	1	4	5	36	757500	14	11
SP11	3	3	2	1	4	6	20A	682500	50	47
SP11	3	3	2	1	4	7	20B			
SP11	3	3	2	1	4	8	24A	1071667	78	75
SP11	3	3	2	1	4	9	24B	56666.67	3	5

experiment	block	trt	CO2	O3	Chamber	SUBREP	flower ID	TTL POL	CT1	CT2
SP11	3	3	2	1	4	10	24C	622500	23	29
SP11	3	3	2	1	4	11	25A			
SP11	3	3	2	1	4	12	25B			
SP11	3	3	2	1	4	13	25C	1357500	61	51
SP11	3	3	2	1	4	14	27A	960000	80	71
SP11	3	3	2	1	4	15	27B			
SP11	3	3	2	1	4	16	27C	1702500	52	59
SP11	3	3	2	1	4	17	33A	716666.7	29	24
SP11	3	3	2	1	4	18	33B	1570000	77	81
SP11	3	3	2	1	4	19	33C	930000	35	29
SP11	3	3	2	1	4	20	33D	875000	29	36
SP11	3	3	2	1	4	21	33E			
SP11	3	3	2	1	4	22	9A			
SP11	3	3	2	1	4	23	9B			
SP11	3	3	2	1	4	24	9C	667500	12	7
SP11	3	3	2	1	4	25	9D	1505000	51	51
SP11	4	2	1	2	5	1	1	692500	65	58
SP11	4	2	1	2	5	2	19	312500	35	30
SP11	4	2	1	2	5	3	20	103333.3	18	23
SP11	4	2	1	2	5	4	22			
SP11	4	2	1	2	5	5	26	152500	10	13
SP11	4	2	1	2	5	6	38	518333.3	92	97
SP11	4	2	1	2	5	7	41	1542500	52	55
SP11	4	2	1	2	5	8	12A	576666.7	25	37
SP11	4	2	1	2	5	9	12B	620000	19	15
SP11	4	2	1	2	5	10	14A	925000	14	46
SP11	4	2	1	2	5	11	14B	897500	53	45

experiment	block	trt	CO2	O3	Chamber	SUBREP	flower ID	TTL POL	CT1	CT2
SP11	4	2	1	2	5	12	14C	725000	18	15
SP11	4	2	1	2	5	13	40A	511666.7	53	43
SP11	4	2	1	2	5	14	40B	777500	34	31
SP11	4	2	1	2	5	15	40C	655000	12	9
SP11	4	2	1	2	5	16	40D	840000	57	66
SP11	4	2	1	2	5	17	40E	185000	5	6
SP11	4	3	2	1	6	1	5	775000	30	33
SP11	4	3	2	1	6	2	28	475000	19	41
SP11	4	3	2	1	6	3	29			
SP11	4	3	2	1	6	4	37	345000	15	17
SP11	4	3	2	1	6	5	25A	837500	21	19
SP11	4	3	2	1	6	6	25B			
SP11	4	3	2	1	6	7	27A	1697500	112	92
SP11	4	3	2	1	6	8	27B	1722500	92	126
SP11	4	3	2	1	6	9	27C	1002500	43	42
SP11	4	3	2	1	6	10	27D			
SP11	4	3	2	1	6	11	27E			
SP11	4	3	2	1	6	12	42A			
SP11	4	3	2	1	6	13	42B	286250	9	26
SP11	4	3	2	1	6	14	42C			
SP11	4	1	1	1	7	1	17	168333.3	2	5
SP11	4	1	1	1	7	2	24	942500	18	16
SP11	4	1	1	1	7	3	27			
SP11	4	1	1	1	7	4	46	765000	21	31
SP11	4	1	1	1	7	5	9A			
SP11	4	1	1	1	7	6	9B			
SP11	4	4	2	2	8	1	6	1290000	36	37

experiment	block	trt	CO2	O3	Chamber	SUBREP	flower ID	TTL POL	CT1	CT2
SP11	4	4	2	2	8	2	12	572500	24	15
SP11	4	4	2	2	8	3	28	565000	11	28
SP11	4	4	2	2	8	4	42	1616667	26	27
SP11	4	4	2	2	8	5	45	907500	19	21
SP11	4	4	2	2	8	6	19A			
SP11	4	4	2	2	8	7	19B	281666.7	11	17
SP11	4	4	2	2	8	8	19C	370000	8	9
SP11	4	4	2	2	8	9	29A	166666.7	1	12
SP11	4	4	2	2	8	10	29B	840000	15	10
SP11	4	4	2	2	8	11	39A			
SP11	4	4	2	2	8	12	39B	420000	6	8
SP11	4	4	2	2	8	13	3A	1655000	76	78
SP11	4	4	2	2	8	14	3B	442500	19	20
SP11	4	4	2	2	8	15	3C	690000	18	22
SP11	4	4	2	2	8	16	3D	1500000	139	94
SP12	5	4	2	2	1	1	1	625000	56	43
SP12	5	4	2	2	1	2	2	850000	31	21
SP12	5	4	2	2	1	3	3	1383750	148	135
SP12	5	4	2	2	1	4	4	1172500	43	42
SP12	5	4	2	2	1	5	5	1312500	109	117
SP12	5	1	1	1	2	1	1	946250	39	44
SP12	5	1	1	1	2	2	2	932500	50	61
SP12	5	1	1	1	2	3	3	490000	27	37
SP12	5	1	1	1	2	4	4	440000	15	24
SP12	5	1	1	1	2	5	5	297500	4	10
SP12	5	3	2	1	3	1	1	1706250	83	73
SP12	5	3	2	1	3	2	2	472500	36	44

experiment	block	trt	CO2	O3	Chamber	SUBREP	flower ID	TTL POL	CT1	CT2
SP12	5	3	2	1	3	3	3	620000	50	58
SP12	5	3	2	1	3	4	4	1237500	95	86
SP12	5	3	2	1	3	5	5	565000	46	53
SP12	5	3	2	1	3	6	6			
SP12	5	3	2	1	3	7	7	925000	85	103
SP12	5	3	2	1	3	8	8			
SP12	5	3	2	1	3	9	9	1642500	55	54
SP12	6	1	1	1	4	1	1	2045000	53	43
SP12	6	1	1	1	4	2	2	636250	29	29
SP12	6	1	1	1	4	3	3	1197500	50	62
SP12	6	1	1	1	4	4	4	810000	20	22
SP12	6	4	2	2	5	1	1	1023750	42	55
SP12	6	4	2	2	5	2	2	1995000	82	68
SP12	6	4	2	2	5	3	3	1620000	82	84
SP12	6	4	2	2	5	4	4	912500	35	37
SP12	6	4	2	2	5	5	5	1385000	44	50
SP12	5	2	1	2	6	1				
SP12	6	3	2	1	7	1	1			
SP12	6	3	2	1	7	2	2			
SP12	6	3	2	1	7	3	3	252500	27	20
SP12	6	3	2	1	7	4	4	2455000	88	97
SP12	5	2	1	2	8	1	1	300000	10	15
SP12	5	2	1	2	8	2	2	230000	14	8

Phleum pratense L.

treatments

experiment	block	trt	CO2	O3	Chamber	SUBREP	flower ID	TTL POL	CT1	CT2
	ambient (1)	elevated (2)			trt 1	trt 2	trt 3			
CO2	400 ppm	800 ppm			30 ppb O3	80 ppb O3	30 ppb O3			
O3	30 ppb	80 ppb			400 ppm CO2	400 ppm C	800 ppm C			
3 experiment	FA11	SP11	SP12							
8 chambers per experiment in a complete factorial design										
2 reps (block) per experiment										
Chamber experiment (CSTR)										
All flowers collected from each chamber										
flower bagged upon emergence and immediately removed and placed in freezer after dehiscence of pc										
Flowers washed with PBS-T (3 washes; pollen removed from each wash via centrifuge; three washes co										
pollen suspended in known volume and counted using hemocytometer (the total amount of pollen fro										
Phl-p-5 content determined using ELISA										
ALL FLOWERS WERE COLLECTED FROM ALL CHAMBERS										
Some flowers were damaged in collection (ie pollen lost) and not used in analysis of pollen content or pent										

								WASH 2		
experiment	block	trt	CO2	O3	Chamber	SUBREP	flower ID	CT3	CT4	AVERAGE
FA11	2	4	2	2	1	1				
FA11	1	2	1	2	2	1	1			59.5
FA11	1	2	1	2	2	2	2			75
FA11	1	2	1	2	2	3	3			91.5
FA11	1	2	1	2	2	4	4			91
FA11	1	3	2	1	3	1	1	65	63	55.5
FA11	1	3	2	1	3	2	2			71
FA11	1	3	2	1	3	3	3			84.5
FA11	1	3	2	1	3	4	4			
FA11	1	3	2	1	3	5	5			
FA11	1	3	2	1	3	6	6			
FA11	1	3	2	1	3	7	7			
FA11	1	3	2	1	3	8	8			94.5
FA11	1	3	2	1	3	9	9	110	112	100.75
FA11	1	3	2	1	3	10	10			88.5
FA11	1	3	2	1	3	11	11			45.5
FA11	1	3	2	1	3	12	12	49	62	64.75
FA11	1	3	2	1	3	13	13			61
FA11	1	3	2	1	3	14	14			98.5
FA11	1	3	2	1	3	15	15			66
FA11	1	1	1	1	4	1	1	74	68	59.75
FA11	1	1	1	1	4	2	2	68	69	62.75
FA11	1	1	1	1	4	3	3			64
FA11	1	1	1	1	4	4	4			94
FA11	1	1	1	1	4	5	5			55.5
FA11	1	1	1	1	4	6	6			89
FA11	1	1	1	1	4	7	7	36	61	48.25

								WASH 2		
experiment	block	trt	CO2	O3	Chamber	SUBREP	flower ID	CT3	CT4	AVERAGE
FA11	1	1	1	1	4	8	8			84
FA11	1	1	1	1	4	9	9	42	49	39.25
FA11	1	1	1	1	4	10	10	105	109	103.5
FA11	1	1	1	1	4	11	11	89	98	97
FA11	1	4	2	2	5	1	1			94
FA11	1	4	2	2	5	2	2			115
FA11	1	4	2	2	5	3	3	56	42	62.25
FA11	1	4	2	2	5	4	4	41		39.66667
FA11	1	4	2	2	5	5	5			71.5
FA11	1	4	2	2	5	6	6			57.5
FA11	1	4	2	2	5	7	7	72	159	131.25
FA11	1	4	2	2	5	8	8			145
FA11	1	4	2	2	5	9	9			
FA11	1	4	2	2	5	10	10			89.5
FA11	1	4	2	2	5	11	11			43
FA11	1	4	2	2	5	12	12	169	122	147.75
FA11	1	4	2	2	5	13	13			51
FA11	1	4	2	2	5	14	14	98	100	106
FA11	1	4	2	2	5	15	15			
FA11	2	1	1	1	6	1	1			75
FA11	2	1	1	1	6	2	2			59.5
FA11	2	3	2	1	7	1	1	82	80	91.75
FA11	2	3	2	1	7	2	2			62.5
FA11	2	3	2	1	7	3	3	24	61	61.75
FA11	2	3	2	1	7	4	4			
FA11	2	3	2	1	7	5	5	125	99	101.75
FA11	2	3	2	1	7	6	6			

								WASH 2		
experiment	block	trt	CO2	O3	Chamber	SUBREP	flower ID	CT3	CT4	AVERAGE
FA11	2	2	1	2	8	1				
SP11	3	2	1	2	1	1	22	0		2
SP11	3	2	1	2	1	2	18A	10		15
SP11	3	2	1	2	1	3	18B			41.5
SP11	3	2	1	2	1	4	24A			41
SP11	3	2	1	2	1	5	24B	33		33.33333
SP11	3	2	1	2	1	6	24C			31.5
SP11	3	2	1	2	1	7	24D			40.5
SP11	3	2	1	2	1	8	24E	8		11
SP11	3	2	1	2	1	9	29A	23		23
SP11	3	2	1	2	1	10	29B	12		8.666667
SP11	3	2	1	2	1	11	32A			34
SP11	3	2	1	2	1	12	32B			
SP11	3	2	1	2	1	13	32C			14
SP11	3	4	2	2	2	1	36	73		70.66667
SP11	3	4	2	2	2	2	40			24
SP11	3	4	2	2	2	3	44	18		14
SP11	3	4	2	2	2	4	23A			72.5
SP11	3	4	2	2	2	5	23B			22.5
SP11	3	4	2	2	2	6	23C			50.5
SP11	3	4	2	2	2	7	29A			21
SP11	3	4	2	2	2	8	29B			25
SP11	3	4	2	2	2	9	2A			20.5
SP11	3	4	2	2	2	10	2B	60		69.66667
SP11	3	4	2	2	2	11	2C	22		21
SP11	3	4	2	2	2	12	38A			35.5
SP11	3	4	2	2	2	13	38B			19

								WASH 2		
experiment	block	trt	CO2	O3	Chamber	SUBREP	flower ID	CT3	CT4	AVERAGE
SP11	3	4	2	2	2	14	38C	11		10.33333
SP11	3	4	2	2	2	15	43A	12		12.33333
SP11	3	4	2	2	2	16	43B			
SP11	3	4	2	2	2	17	43C			93
SP11	3	4	2	2	2	18	43D			46
SP11	3	4	2	2	2	19	43E	31		34
SP11	3	4	2	2	2	20	45A			21
SP11	3	4	2	2	2	21	45B			53
SP11	3	4	2	2	2	22	45C			37
SP11	3	1	1	1	3	1	6			12
SP11	3	1	1	1	3	2	27			19
SP11	3	1	1	1	3	3	37			
SP11	3	1	1	1	3	4	41	9		7.333333
SP11	3	1	1	1	3	5	29A	18		16.33333
SP11	3	1	1	1	3	6	29B			36.5
SP11	3	1	1	1	3	7	29C			17
SP11	3	1	1	1	3	8	9A			19.5
SP11	3	1	1	1	3	9	9B			28.5
SP11	3	3	2	1	4	1	16			
SP11	3	3	2	1	4	2	17	71		70
SP11	3	3	2	1	4	3	30			23.5
SP11	3	3	2	1	4	4	31			
SP11	3	3	2	1	4	5	36			12.5
SP11	3	3	2	1	4	6	20A			48.5
SP11	3	3	2	1	4	7	20B			
SP11	3	3	2	1	4	8	24A	76		76.33333
SP11	3	3	2	1	4	9	24B	7		5

								WASH 2		
experiment	block	trt	CO2	O3	Chamber	SUBREP	flower ID	CT3	CT4	AVERAGE
SP11	3	3	2	1	4	10	24C			26
SP11	3	3	2	1	4	11	25A			
SP11	3	3	2	1	4	12	25B			
SP11	3	3	2	1	4	13	25C			56
SP11	3	3	2	1	4	14	27A			75.5
SP11	3	3	2	1	4	15	27B			
SP11	3	3	2	1	4	16	27C			55.5
SP11	3	3	2	1	4	17	33A	38		30.33333
SP11	3	3	2	1	4	18	33B			79
SP11	3	3	2	1	4	19	33C			32
SP11	3	3	2	1	4	20	33D			32.5
SP11	3	3	2	1	4	21	33E			
SP11	3	3	2	1	4	22	9A			
SP11	3	3	2	1	4	23	9B			
SP11	3	3	2	1	4	24	9C			9.5
SP11	3	3	2	1	4	25	9D			51
SP11	4	2	1	2	5	1	1	84		69
SP11	4	2	1	2	5	2	19			32.5
SP11	4	2	1	2	5	3	20	26		22.33333
SP11	4	2	1	2	5	4	22			
SP11	4	2	1	2	5	5	26			11.5
SP11	4	2	1	2	5	6	38	74		87.66667
SP11	4	2	1	2	5	7	41			53.5
SP11	4	2	1	2	5	8	12A	30		30.66667
SP11	4	2	1	2	5	9	12B			17
SP11	4	2	1	2	5	10	14A	49	39	37
SP11	4	2	1	2	5	11	14B			49

								WASH 2		
experiment	block	trt	CO2	O3	Chamber	SUBREP	flower ID	CT3	CT4	AVERAGE
SP11	4	2	1	2	5	12	14C			16.5
SP11	4	2	1	2	5	13	40A	36		44
SP11	4	2	1	2	5	14	40B			32.5
SP11	4	2	1	2	5	15	40C			10.5
SP11	4	2	1	2	5	16	40D	48	59	57.5
SP11	4	2	1	2	5	17	40E	8		6.333333
SP11	4	3	2	1	6	1	5			31.5
SP11	4	3	2	1	6	2	28	38		32.66667
SP11	4	3	2	1	6	3	29			
SP11	4	3	2	1	6	4	37	15		15.66667
SP11	4	3	2	1	6	5	25A			20
SP11	4	3	2	1	6	6	25B			
SP11	4	3	2	1	6	7	27A			102
SP11	4	3	2	1	6	8	27B	79		99
SP11	4	3	2	1	6	9	27C			42.5
SP11	4	3	2	1	6	10	27D			
SP11	4	3	2	1	6	11	27E			
SP11	4	3	2	1	6	12	42A			
SP11	4	3	2	1	6	13	42B	21		18.66667
SP11	4	3	2	1	6	14	42C			
SP11	4	1	1	1	7	1	17	4		3.666667
SP11	4	1	1	1	7	2	24			17
SP11	4	1	1	1	7	3	27			
SP11	4	1	1	1	7	4	46			26
SP11	4	1	1	1	7	5	9A			
SP11	4	1	1	1	7	6	9B			
SP11	4	4	2	2	8	1	6			36.5

								WASH 2		
experiment	block	trt	CO2	O3	Chamber	SUBREP	flower ID	CT3	CT4	AVERAGE
SP11	4	4	2	2	8	2	12			19.5
SP11	4	4	2	2	8	3	28	43	32	28.5
SP11	4	4	2	2	8	4	42	36		29.66667
SP11	4	4	2	2	8	5	45	22		20.66667
SP11	4	4	2	2	8	6	19A			
SP11	4	4	2	2	8	7	19B	13		13.66667
SP11	4	4	2	2	8	8	19C	7		8
SP11	4	4	2	2	8	9	29A	6		6.333333
SP11	4	4	2	2	8	10	29B			12.5
SP11	4	4	2	2	8	11	39A			
SP11	4	4	2	2	8	12	39B	5		6.333333
SP11	4	4	2	2	8	13	3A			77
SP11	4	4	2	2	8	14	3B			19.5
SP11	4	4	2	2	8	15	3C			20
SP11	4	4	2	2	8	16	3D			116.5
SP12	5	4	2	2	1	1	1	37	61	49.25
SP12	5	4	2	2	1	2	2			26
SP12	5	4	2	2	1	3	3			141.5
SP12	5	4	2	2	1	4	4			42.5
SP12	5	4	2	2	1	5	5			113
SP12	5	1	1	1	2	1	1			41.5
SP12	5	1	1	1	2	2	2			55.5
SP12	5	1	1	1	2	3	3			32
SP12	5	1	1	1	2	4	4	30	21	22.5
SP12	5	1	1	1	2	5	5			7
SP12	5	3	2	1	3	1	1			78
SP12	5	3	2	1	3	2	2			40

								WASH 2		
experiment	block	trt	CO2	O3	Chamber	SUBREP	flower ID	CT3	CT4	AVERAGE
SP12	5	3	2	1	3	3	3	32	35	43.75
SP12	5	3	2	1	3	4	4			90.5
SP12	5	3	2	1	3	5	5			49.5
SP12	5	3	2	1	3	6	6			
SP12	5	3	2	1	3	7	7	82	117	96.75
SP12	5	3	2	1	3	8	8			
SP12	5	3	2	1	3	9	9			54.5
SP12	6	1	1	1	4	1	1	28	33	39.25
SP12	6	1	1	1	4	2	2			29
SP12	6	1	1	1	4	3	3			56
SP12	6	1	1	1	4	4	4			21
SP12	6	4	2	2	5	1	1			48.5
SP12	6	4	2	2	5	2	2	63	82	73.75
SP12	6	4	2	2	5	3	3			83
SP12	6	4	2	2	5	4	4			36
SP12	6	4	2	2	5	5	5	83	81	64.5
SP12	5	2	1	2	6	1				
SP12	6	3	2	1	7	1	1			
SP12	6	3	2	1	7	2	2			
SP12	6	3	2	1	7	3	3			23.5
SP12	6	3	2	1	7	4	4			92.5
SP12	5	2	1	2	8	1	1			12.5
SP12	5	2	1	2	8	2	2			11

Phleum pratense L.

treatments

								WASH 2		
experiment	block	trt	CO2	O3	Chamber	SUBREP	flower ID	CT3	CT4	AVERAGE
	ambient (1)	elevated (2)			trt 1	trt 2	trt 3			
CO2	400 ppm	800 ppm			30 ppb O3	80 ppb O3	30 ppb O3			
O3	30 ppb	80 ppb			400 ppm CO2	400 ppm C	800 ppm C			
3 experiment	FA11	SP11	SP12							
8 chambers per experiment in a complete factorial design										
2 reps (block) per experiment										
Chamber experiment (CSTR)										
All flowers collected from each chamber										
flower bagged upon emergence and immediately removed and placed in freezer after dehiscence of po										
Flowers washed with PBS-T (3 washes; pollen removed from each wash via centrifuge; three washes co										
pollen suspended in known volume and counted using hemocytometer (the total amount of pollen fro										
Phl-p-5 content determined using ELISA										
ALL FLOWERS WERE COLLECTED FROM ALL CHAMBERS										
Some flowers were damaged in collection (ie pollen lost) and not used in analysis of pollen content or p										

experiment	block	trt	CO2	O3	Chamber	SUBREP	flower ID	WASH UL	TTL POL	CT1
FA11	2	4	2	2	1	1				
FA11	1	2	1	2	2	1	1	500	297500	9
FA11	1	2	1	2	2	2	2	1000	750000	95
FA11	1	2	1	2	2	3	3	1000	915000	70
FA11	1	2	1	2	2	4	4	1000	910000	51
FA11	1	3	2	1	3	1	1	500	277500	10
FA11	1	3	2	1	3	2	2	1000	710000	61
FA11	1	3	2	1	3	3	3	1000	845000	97
FA11	1	3	2	1	3	4	4			
FA11	1	3	2	1	3	5	5			
FA11	1	3	2	1	3	6	6			
FA11	1	3	2	1	3	7	7			
FA11	1	3	2	1	3	8	8	1500	1417500	35
FA11	1	3	2	1	3	9	9	500	503750	29
FA11	1	3	2	1	3	10	10	1000	885000	86
FA11	1	3	2	1	3	11	11	500	227500	18
FA11	1	3	2	1	3	12	12	500	323750	18
FA11	1	3	2	1	3	13	13	1500	915000	58
FA11	1	3	2	1	3	14	14	1000	985000	99
FA11	1	3	2	1	3	15	15	1000	660000	86
FA11	1	1	1	1	4	1	1	500	298750	17
FA11	1	1	1	1	4	2	2	500	313750	37
FA11	1	1	1	1	4	3	3	500	320000	29
FA11	1	1	1	1	4	4	4	1000	940000	64
FA11	1	1	1	1	4	5	5	500	277500	36
FA11	1	1	1	1	4	6	6	500	445000	42
FA11	1	1	1	1	4	7	7	500	241250	42

experiment	block	trt	CO2	O3	Chamber	SUBREP	flower ID	WASH UL	TTL POL	CT1
FA11	1	1	1	1	4	8	8	500	420000	44
FA11	1	1	1	1	4	9	9	500	196250	18
FA11	1	1	1	1	4	10	10	1000	1035000	72
FA11	1	1	1	1	4	11	11	1000	970000	63
FA11	1	4	2	2	5	1	1	1500	1410000	72
FA11	1	4	2	2	5	2	2	1000	1150000	88
FA11	1	4	2	2	5	3	3	1000	622500	44
FA11	1	4	2	2	5	4	4	1000	396666.7	28
FA11	1	4	2	2	5	5	5	1500	1072500	63
FA11	1	4	2	2	5	6	6	500	287500	15
FA11	1	4	2	2	5	7	7	500	656250	28
FA11	1	4	2	2	5	8	8	1000	1450000	125
FA11	1	4	2	2	5	9	9			
FA11	1	4	2	2	5	10	10	1000	895000	51
FA11	1	4	2	2	5	11	11	1000	430000	59
FA11	1	4	2	2	5	12	12	500	738750	110
FA11	1	4	2	2	5	13	13	500	255000	17
FA11	1	4	2	2	5	14	14	1000	1060000	74
FA11	1	4	2	2	5	15	15			
FA11	2	1	1	1	6	1	1	500	375000	31
FA11	2	1	1	1	6	2	2	500	297500	38
FA11	2	3	2	1	7	1	1	1000	917500	70
FA11	2	3	2	1	7	2	2	500	312500	14
FA11	2	3	2	1	7	3	3	1000	617500	67
FA11	2	3	2	1	7	4	4			
FA11	2	3	2	1	7	5	5	1000	1017500	79
FA11	2	3	2	1	7	6	6			

experiment	block	trt	CO2	O3	Chamber	SUBREP	flower ID	WASH UL	TTL POL	CT1
FA11	2	2	1	2	8	1				
SP11	3	2	1	2	1	1	22	500	10000	0
SP11	3	2	1	2	1	2	18A	500	75000	0
SP11	3	2	1	2	1	3	18B	500	207500	4
SP11	3	2	1	2	1	4	24A	500	205000	10
SP11	3	2	1	2	1	5	24B	500	166666.7	0
SP11	3	2	1	2	1	6	24C	500	157500	13
SP11	3	2	1	2	1	7	24D	500	202500	8
SP11	3	2	1	2	1	8	24E	500	55000	0
SP11	3	2	1	2	1	9	29A	500	115000	0
SP11	3	2	1	2	1	10	29B	500	43333.33	0
SP11	3	2	1	2	1	11	32A	500	170000	9
SP11	3	2	1	2	1	12	32B			
SP11	3	2	1	2	1	13	32C	500	70000	4
SP11	3	4	2	2	2	1	36	500	353333.3	0
SP11	3	4	2	2	2	2	40	500	120000	8
SP11	3	4	2	2	2	3	44	500	70000	0
SP11	3	4	2	2	2	4	23A	500	362500	17
SP11	3	4	2	2	2	5	23B	500	112500	6
SP11	3	4	2	2	2	6	23C	500	252500	46
SP11	3	4	2	2	2	7	29A	500	105000	5
SP11	3	4	2	2	2	8	29B	500	125000	7
SP11	3	4	2	2	2	9	2A	500	102500	10
SP11	3	4	2	2	2	10	2B	500	348333.3	0
SP11	3	4	2	2	2	11	2C	500	105000	0
SP11	3	4	2	2	2	12	38A	500	177500	13
SP11	3	4	2	2	2	13	38B	500	95000	3

experiment	block	trt	CO2	O3	Chamber	SUBREP	flower ID	WASH UL	TTL POL	CT1
SP11	3	4	2	2	2	14	38C	500	51666.67	0
SP11	3	4	2	2	2	15	43A	500	61666.67	0
SP11	3	4	2	2	2	16	43B			
SP11	3	4	2	2	2	17	43C	500	465000	4
SP11	3	4	2	2	2	18	43D	500	230000	10
SP11	3	4	2	2	2	19	43E	500	170000	0
SP11	3	4	2	2	2	20	45A	500	105000	8
SP11	3	4	2	2	2	21	45B	500	265000	24
SP11	3	4	2	2	2	22	45C	500	185000	7
SP11	3	1	1	1	3	1	6	500	60000	3
SP11	3	1	1	1	3	2	27	500	95000	4
SP11	3	1	1	1	3	3	37			
SP11	3	1	1	1	3	4	41	500	36666.67	0
SP11	3	1	1	1	3	5	29A	500	81666.67	0
SP11	3	1	1	1	3	6	29B	500	182500	8
SP11	3	1	1	1	3	7	29C	500	85000	1
SP11	3	1	1	1	3	8	9A	500	97500	3
SP11	3	1	1	1	3	9	9B	500	142500	3
SP11	3	3	2	1	4	1	16			
SP11	3	3	2	1	4	2	17	500	350000	0
SP11	3	3	2	1	4	3	30	500	117500	7
SP11	3	3	2	1	4	4	31			
SP11	3	3	2	1	4	5	36	500	62500	6
SP11	3	3	2	1	4	6	20A	500	242500	12
SP11	3	3	2	1	4	7	20B			
SP11	3	3	2	1	4	8	24A	500	381666.7	0
SP11	3	3	2	1	4	9	24B	500	25000	0

experiment	block	trt	CO2	O3	Chamber	SUBREP	flower ID	WASH UL	TTL POL	CT1
SP11	3	3	2	1	4	10	24C	500	130000	1
SP11	3	3	2	1	4	11	25A			
SP11	3	3	2	1	4	12	25B			
SP11	3	3	2	1	4	13	25C	500	280000	28
SP11	3	3	2	1	4	14	27A	500	377500	36
SP11	3	3	2	1	4	15	27B			
SP11	3	3	2	1	4	16	27C	500	277500	21
SP11	3	3	2	1	4	17	33A	500	151666.7	0
SP11	3	3	2	1	4	18	33B	500	395000	25
SP11	3	3	2	1	4	19	33C	500	160000	8
SP11	3	3	2	1	4	20	33D	500	162500	7
SP11	3	3	2	1	4	21	33E			
SP11	3	3	2	1	4	22	9A			
SP11	3	3	2	1	4	23	9B			
SP11	3	3	2	1	4	24	9C	500	47500	1
SP11	3	3	2	1	4	25	9D	500	255000	10
SP11	4	2	1	2	5	1	1	500	345000	0
SP11	4	2	1	2	5	2	19	500	162500	10
SP11	4	2	1	2	5	3	20	500	111666.7	0
SP11	4	2	1	2	5	4	22			
SP11	4	2	1	2	5	5	26	500	57500	13
SP11	4	2	1	2	5	6	38	500	438333.3	0
SP11	4	2	1	2	5	7	41	500	267500	18
SP11	4	2	1	2	5	8	12A	500	153333.3	0
SP11	4	2	1	2	5	9	12B	500	85000	42
SP11	4	2	1	2	5	10	14A	500	185000	0
SP11	4	2	1	2	5	11	14B	500	245000	13

experiment	block	trt	CO2	O3	Chamber	SUBREP	flower ID	WASH UL	TTL POL	CT1
SP11	4	2	1	2	5	12	14C	500	82500	4
SP11	4	2	1	2	5	13	40A	500	220000	0
SP11	4	2	1	2	5	14	40B	500	162500	4
SP11	4	2	1	2	5	15	40C	500	52500	6
SP11	4	2	1	2	5	16	40D	500	287500	0
SP11	4	2	1	2	5	17	40E	500	31666.67	0
SP11	4	3	2	1	6	1	5	500	157500	15
SP11	4	3	2	1	6	2	28	500	163333.3	0
SP11	4	3	2	1	6	3	29			
SP11	4	3	2	1	6	4	37	500	78333.33	0
SP11	4	3	2	1	6	5	25A	500	100000	5
SP11	4	3	2	1	6	6	25B			
SP11	4	3	2	1	6	7	27A	500	510000	33
SP11	4	3	2	1	6	8	27B	500	495000	21
SP11	4	3	2	1	6	9	27C	500	212500	4
SP11	4	3	2	1	6	10	27D			
SP11	4	3	2	1	6	11	27E			
SP11	4	3	2	1	6	12	42A			
SP11	4	3	2	1	6	13	42B	500	93333.33	0
SP11	4	3	2	1	6	14	42C			
SP11	4	1	1	1	7	1	17	500	18333.33	0
SP11	4	1	1	1	7	2	24	500	85000	9
SP11	4	1	1	1	7	3	27			
SP11	4	1	1	1	7	4	46	500	130000	7
SP11	4	1	1	1	7	5	9A			
SP11	4	1	1	1	7	6	9B			
SP11	4	4	2	2	8	1	6	500	182500	17

experiment	block	trt	CO2	O3	Chamber	SUBREP	flower ID	WASH UL	TTL POL	CT1
SP11	4	4	2	2	8	2	12	500	97500	11
SP11	4	4	2	2	8	3	28	500	142500	0
SP11	4	4	2	2	8	4	42	500	148333.3	0
SP11	4	4	2	2	8	5	45	500	103333.3	0
SP11	4	4	2	2	8	6	19A			
SP11	4	4	2	2	8	7	19B	500	68333.33	0
SP11	4	4	2	2	8	8	19C	500	40000	0
SP11	4	4	2	2	8	9	29A	500	31666.67	0
SP11	4	4	2	2	8	10	29B	500	62500	3
SP11	4	4	2	2	8	11	39A			
SP11	4	4	2	2	8	12	39B	500	31666.67	0
SP11	4	4	2	2	8	13	3A	500	385000	13
SP11	4	4	2	2	8	14	3B	500	97500	3
SP11	4	4	2	2	8	15	3C	500	100000	6
SP11	4	4	2	2	8	16	3D	500	582500	51
SP12	5	4	2	2	1	1	1	500	246250	29
SP12	5	4	2	2	1	2	2	500	130000	10
SP12	5	4	2	2	1	3	3	500	707500	27
SP12	5	4	2	2	1	4	4	500	212500	16
SP12	5	4	2	2	1	5	5	500	565000	53
SP12	5	1	1	1	2	1	1	500	207500	27
SP12	5	1	1	1	2	2	2	500	277500	9
SP12	5	1	1	1	2	3	3	500	160000	11
SP12	5	1	1	1	2	4	4	500	112500	144
SP12	5	1	1	1	2	5	5	500	35000	4
SP12	5	3	2	1	3	1	1	1000	780000	47
SP12	5	3	2	1	3	2	2	500	200000	15

experiment	block	trt	CO2	O3	Chamber	SUBREP	flower ID	WASH UL	TTL POL	CT1
SP12	5	3	2	1	3	3	3	500	218750	18
SP12	5	3	2	1	3	4	4	500	452500	26
SP12	5	3	2	1	3	5	5	500	247500	36
SP12	5	3	2	1	3	6	6			
SP12	5	3	2	1	3	7	7	500	483750	60
SP12	5	3	2	1	3	8	8			
SP12	5	3	2	1	3	9	9	500	272500	19
SP12	6	1	1	1	4	1	1	500	196250	12
SP12	6	1	1	1	4	2	2	500	145000	9
SP12	6	1	1	1	4	3	3	500	280000	11
SP12	6	1	1	1	4	4	4	500	105000	14
SP12	6	4	2	2	5	1	1	500	242500	31
SP12	6	4	2	2	5	2	2	500	368750	17
SP12	6	4	2	2	5	3	3	500	415000	28
SP12	6	4	2	2	5	4	4	500	180000	8
SP12	6	4	2	2	5	5	5	500	322500	30
SP12	5	2	1	2	6	1				
SP12	6	3	2	1	7	1	1			
SP12	6	3	2	1	7	2	2			
SP12	6	3	2	1	7	3	3	500	117500	5
SP12	6	3	2	1	7	4	4	500	462500	69
SP12	5	2	1	2	8	1	1	500	62500	32
SP12	5	2	1	2	8	2	2	500	55000	7

Phleum pratense L.

treatments

experiment	block	trt	CO2	O3	Chamber	SUBREP	flower ID	WASH UL	TTL POL	CT1
	ambient (1)	elevated (2)			trt 1	trt 2	trt 3			
CO2	400 ppm	800 ppm			30 ppb O3	80 ppb O3	30 ppb O3			
O3	30 ppb	80 ppb			400 ppm CO2	400 ppm C	800 ppm C			
3 experiment	FA11	SP11	SP12							
8 chambers per experiment in a complete factorial design										
2 reps (block) per experiment										
Chamber experiment (CSTR)										
All flowers collected from each chamber										
flower bagged upon emergence and immediately removed and placed in freezer after dehiscence of po										
Flowers washed with PBS-T (3 washes; pollen removed from each wash via centrifuge; three washes co										
pollen suspended in known volume and counted using hemocytometer (the total amount of pollen fro										
Phl-p-5 content determined using ELISA										
ALL FLOWERS WERE COLLECTED FROM ALL CHAMBERS										
Some flowers were damaged in collection (ie pollen lost) and not used in analysis of pollen content or p										

								WASH 3		
experiment	block	trt	CO2	O3	Chamber	SUBREP	flower ID	CT2	CT3	CT4
FA11	2	4	2	2	1	1				
FA11	1	2	1	2	2	1	1	15		
FA11	1	2	1	2	2	2	2	85		
FA11	1	2	1	2	2	3	3	61	95	77
FA11	1	2	1	2	2	4	4	42		
FA11	1	3	2	1	3	1	1	12		
FA11	1	3	2	1	3	2	2	63	83	80
FA11	1	3	2	1	3	3	3	64	65	81
FA11	1	3	2	1	3	4	4			
FA11	1	3	2	1	3	5	5			
FA11	1	3	2	1	3	6	6			
FA11	1	3	2	1	3	7	7			
FA11	1	3	2	1	3	8	8	56	56	57
FA11	1	3	2	1	3	9	9	27		
FA11	1	3	2	1	3	10	10	87	68	88
FA11	1	3	2	1	3	11	11	19		
FA11	1	3	2	1	3	12	12	23		
FA11	1	3	2	1	3	13	13	92	80	93
FA11	1	3	2	1	3	14	14	75	78	146
FA11	1	3	2	1	3	15	15	69	61	68
FA11	1	1	1	1	4	1	1	21		
FA11	1	1	1	1	4	2	2	43		
FA11	1	1	1	1	4	3	3	39		
FA11	1	1	1	1	4	4	4	54		
FA11	1	1	1	1	4	5	5	41		
FA11	1	1	1	1	4	6	6	42		
FA11	1	1	1	1	4	7	7	39		

										WASH 3
experiment	block	trt	CO2	O3	Chamber	SUBREP	flower ID	CT2	CT3	CT4
FA11	1	1	1	1	4	8	8	52		
FA11	1	1	1	1	4	9	9	42	34	35
FA11	1	1	1	1	4	10	10	58		
FA11	1	1	1	1	4	11	11	62	95	52
FA11	1	4	2	2	5	1	1	67		
FA11	1	4	2	2	5	2	2	95		
FA11	1	4	2	2	5	3	3	36		
FA11	1	4	2	2	5	4	4	28		
FA11	1	4	2	2	5	5	5	65	110	79
FA11	1	4	2	2	5	6	6	13		
FA11	1	4	2	2	5	7	7	30		
FA11	1	4	2	2	5	8	8	102	102	114
FA11	1	4	2	2	5	9	9			
FA11	1	4	2	2	5	10	10	46		
FA11	1	4	2	2	5	11	11	50		
FA11	1	4	2	2	5	12	12	106	91	88
FA11	1	4	2	2	5	13	13	21		
FA11	1	4	2	2	5	14	14	79		
FA11	1	4	2	2	5	15	15			
FA11	2	1	1	1	6	1	1	30		
FA11	2	1	1	1	6	2	2	30		
FA11	2	3	2	1	7	1	1	60		
FA11	2	3	2	1	7	2	2	16		
FA11	2	3	2	1	7	3	3	72		
FA11	2	3	2	1	7	4	4			
FA11	2	3	2	1	7	5	5	106	91	82
FA11	2	3	2	1	7	6	6			

										WASH 3
experiment	block	trt	CO2	O3	Chamber	SUBREP	flower ID	CT2	CT3	CT4
FA11	2	2	1	2	8	1				
SP11	3	2	1	2	1	1	22			
SP11	3	2	1	2	1	2	18A			
SP11	3	2	1	2	1	3	18B	4		
SP11	3	2	1	2	1	4	24A	16		
SP11	3	2	1	2	1	5	24B			
SP11	3	2	1	2	1	6	24C	15		
SP11	3	2	1	2	1	7	24D	9		
SP11	3	2	1	2	1	8	24E			
SP11	3	2	1	2	1	9	29A			
SP11	3	2	1	2	1	10	29B			
SP11	3	2	1	2	1	11	32A	15		
SP11	3	2	1	2	1	12	32B			
SP11	3	2	1	2	1	13	32C	1		
SP11	3	4	2	2	2	1	36			
SP11	3	4	2	2	2	2	40	9		
SP11	3	4	2	2	2	3	44			
SP11	3	4	2	2	2	4	23A	23		
SP11	3	4	2	2	2	5	23B	4		
SP11	3	4	2	2	2	6	23C	50		
SP11	3	4	2	2	2	7	29A	5		
SP11	3	4	2	2	2	8	29B	7		
SP11	3	4	2	2	2	9	2A	10		
SP11	3	4	2	2	2	10	2B			
SP11	3	4	2	2	2	11	2C			
SP11	3	4	2	2	2	12	38A	7		
SP11	3	4	2	2	2	13	38B	5		

								WASH 3		
experiment	block	trt	CO2	O3	Chamber	SUBREP	flower ID	CT2	CT3	CT4
SP11	3	4	2	2	2	14	38C			
SP11	3	4	2	2	2	15	43A			
SP11	3	4	2	2	2	16	43B			
SP11	3	4	2	2	2	17	43C	4		
SP11	3	4	2	2	2	18	43D	18		
SP11	3	4	2	2	2	19	43E			
SP11	3	4	2	2	2	20	45A	9		
SP11	3	4	2	2	2	21	45B	15		
SP11	3	4	2	2	2	22	45C	12		
SP11	3	1	1	1	3	1	6	2		
SP11	3	1	1	1	3	2	27	2		
SP11	3	1	1	1	3	3	37			
SP11	3	1	1	1	3	4	41			
SP11	3	1	1	1	3	5	29A			
SP11	3	1	1	1	3	6	29B	3		
SP11	3	1	1	1	3	7	29C	2		
SP11	3	1	1	1	3	8	9A	8		
SP11	3	1	1	1	3	9	9B	3		
SP11	3	3	2	1	4	1	16			
SP11	3	3	2	1	4	2	17			
SP11	3	3	2	1	4	3	30	5		
SP11	3	3	2	1	4	4	31			
SP11	3	3	2	1	4	5	36	3		
SP11	3	3	2	1	4	6	20A	14		
SP11	3	3	2	1	4	7	20B			
SP11	3	3	2	1	4	8	24A			
SP11	3	3	2	1	4	9	24B			

								WASH 3		
experiment	block	trt	CO2	O3	Chamber	SUBREP	flower ID	CT2	CT3	CT4
SP11	3	3	2	1	4	10	24C	3		
SP11	3	3	2	1	4	11	25A			
SP11	3	3	2	1	4	12	25B			
SP11	3	3	2	1	4	13	25C	17		
SP11	3	3	2	1	4	14	27A	34		
SP11	3	3	2	1	4	15	27B			
SP11	3	3	2	1	4	16	27C	23		
SP11	3	3	2	1	4	17	33A			
SP11	3	3	2	1	4	18	33B	26		
SP11	3	3	2	1	4	19	33C	8		
SP11	3	3	2	1	4	20	33D	11		
SP11	3	3	2	1	4	21	33E			
SP11	3	3	2	1	4	22	9A			
SP11	3	3	2	1	4	23	9B			
SP11	3	3	2	1	4	24	9C	4		
SP11	3	3	2	1	4	25	9D	13		
SP11	4	2	1	2	5	1	1			
SP11	4	2	1	2	5	2	19	9		
SP11	4	2	1	2	5	3	20			
SP11	4	2	1	2	5	4	22			
SP11	4	2	1	2	5	5	26	4		
SP11	4	2	1	2	5	6	38			
SP11	4	2	1	2	5	7	41	10		
SP11	4	2	1	2	5	8	12A			
SP11	4	2	1	2	5	9	12B	61		
SP11	4	2	1	2	5	10	14A			
SP11	4	2	1	2	5	11	14B	11		

										WASH 3
experiment	block	trt	CO2	O3	Chamber	SUBREP	flower ID	CT2	CT3	CT4
SP11	4	2	1	2	5	12	14C	5		
SP11	4	2	1	2	5	13	40A			
SP11	4	2	1	2	5	14	40B	4		
SP11	4	2	1	2	5	15	40C	1		
SP11	4	2	1	2	5	16	40D			
SP11	4	2	1	2	5	17	40E			
SP11	4	3	2	1	6	1	5	13		
SP11	4	3	2	1	6	2	28			
SP11	4	3	2	1	6	3	29			
SP11	4	3	2	1	6	4	37			
SP11	4	3	2	1	6	5	25A	4		
SP11	4	3	2	1	6	6	25B			
SP11	4	3	2	1	6	7	27A	28		
SP11	4	3	2	1	6	8	27B	26		
SP11	4	3	2	1	6	9	27C	9		
SP11	4	3	2	1	6	10	27D			
SP11	4	3	2	1	6	11	27E			
SP11	4	3	2	1	6	12	42A			
SP11	4	3	2	1	6	13	42B			
SP11	4	3	2	1	6	14	42C			
SP11	4	1	1	1	7	1	17	0		
SP11	4	1	1	1	7	2	24	7		
SP11	4	1	1	1	7	3	27			
SP11	4	1	1	1	7	4	46	10		
SP11	4	1	1	1	7	5	9A			
SP11	4	1	1	1	7	6	9B			
SP11	4	4	2	2	8	1	6	11		

										WASH 3
experiment	block	trt	CO2	O3	Chamber	SUBREP	flower ID	CT2	CT3	CT4
SP11	4	4	2	2	8	2	12	6		
SP11	4	4	2	2	8	3	28			
SP11	4	4	2	2	8	4	42			
SP11	4	4	2	2	8	5	45			
SP11	4	4	2	2	8	6	19A			
SP11	4	4	2	2	8	7	19B			
SP11	4	4	2	2	8	8	19C			
SP11	4	4	2	2	8	9	29A			
SP11	4	4	2	2	8	10	29B	3		
SP11	4	4	2	2	8	11	39A			
SP11	4	4	2	2	8	12	39B			
SP11	4	4	2	2	8	13	3A	28		
SP11	4	4	2	2	8	14	3B	4		
SP11	4	4	2	2	8	15	3C	10		
SP11	4	4	2	2	8	16	3D	38		
SP12	5	4	2	2	1	1	1	21		
SP12	5	4	2	2	1	2	2	13		
SP12	5	4	2	2	1	3	3	17		
SP12	5	4	2	2	1	4	4	10		
SP12	5	4	2	2	1	5	5	56		
SP12	5	1	1	1	2	1	1	28		
SP12	5	1	1	1	2	2	2	11	30	20
SP12	5	1	1	1	2	3	3	4		
SP12	5	1	1	1	2	4	4	133		
SP12	5	1	1	1	2	5	5	3		
SP12	5	3	2	1	3	1	1	41		
SP12	5	3	2	1	3	2	2	28	30	23

								WASH 3		
experiment	block	trt	CO2	O3	Chamber	SUBREP	flower ID	CT2	CT3	CT4
SP12	5	3	2	1	3	3	3	20		
SP12	5	3	2	1	3	4	4	25		
SP12	5	3	2	1	3	5	5	40		
SP12	5	3	2	1	3	6	6			
SP12	5	3	2	1	3	7	7	51		
SP12	5	3	2	1	3	8	8			
SP12	5	3	2	1	3	9	9	10		
SP12	6	1	1	1	4	1	1	16		
SP12	6	1	1	1	4	2	2	10		
SP12	6	1	1	1	4	3	3	13		
SP12	6	1	1	1	4	4	4	10		
SP12	6	4	2	2	5	1	1	22		
SP12	6	4	2	2	5	2	2	16	25	
SP12	6	4	2	2	5	3	3	34		
SP12	6	4	2	2	5	4	4	9		
SP12	6	4	2	2	5	5	5	23		
SP12	5	2	1	2	6	1				
SP12	6	3	2	1	7	1	1			
SP12	6	3	2	1	7	2	2			
SP12	6	3	2	1	7	3	3	6		
SP12	6	3	2	1	7	4	4	61		
SP12	5	2	1	2	8	1	1	26	9	21
SP12	5	2	1	2	8	2	2	2		
Phleum pratense L.										
treatments										

										WASH 3
experiment	block	trt	CO2	O3	Chamber	SUBREP	flower ID	CT2	CT3	CT4
	ambient (1)	elevated (2)			trt 1	trt 2	trt 3			
CO2	400 ppm	800 ppm			30 ppb O3	80 ppb O3	30 ppb O3			
O3	30 ppb	80 ppb			400 ppm CO2	400 ppm C	800 ppm C			
3 experiment	FA11	SP11	SP12							
8 chambers per experiment in a complete factorial design										
2 reps (block) per experiment										
Chamber experiment (CSTR)										
All flowers collected from each chamber										
flower bagged upon emergence and immediately removed and placed in freezer after dehiscence of po										
Flowers washed with PBS-T (3 washes; pollen removed from each wash via centrifuge; three washes co										
pollen suspended in known volume and counted using hemocytometer (the total amount of pollen fro										
Phl-p-5 content determined using ELISA										
ALL FLOWERS WERE COLLECTED FROM ALL CHAMBERS										
Some flowers were damaged in collection (ie pollen lost) and not used in analysis of pollen content or p										

experiment	block	trt	CO2	O3	Chamber	SUBREP	flower ID	AVERAGE	suspensior	TTL POLL
FA11	2	4	2	2	1	1				
FA11	1	2	1	2	2	1	1	12	500	60000
FA11	1	2	1	2	2	2	2	90	500	450000
FA11	1	2	1	2	2	3	3	75.75	500	378750
FA11	1	2	1	2	2	4	4	46.5	500	232500
FA11	1	3	2	1	3	1	1	11	500	55000
FA11	1	3	2	1	3	2	2	71.75	500	358750
FA11	1	3	2	1	3	3	3	76.75	500	383750
FA11	1	3	2	1	3	4	4			
FA11	1	3	2	1	3	5	5			
FA11	1	3	2	1	3	6	6			
FA11	1	3	2	1	3	7	7			
FA11	1	3	2	1	3	8	8	51	1000	510000
FA11	1	3	2	1	3	9	9	28	500	140000
FA11	1	3	2	1	3	10	10	82.25	1000	822500
FA11	1	3	2	1	3	11	11	18.5	500	92500
FA11	1	3	2	1	3	12	12	20.5	500	102500
FA11	1	3	2	1	3	13	13	80.75	500	403750
FA11	1	3	2	1	3	14	14	99.5	500	497500
FA11	1	3	2	1	3	15	15	71	500	355000
FA11	1	1	1	1	4	1	1	19	500	95000
FA11	1	1	1	1	4	2	2	40	500	200000
FA11	1	1	1	1	4	3	3	34	500	170000
FA11	1	1	1	1	4	4	4	59	500	295000
FA11	1	1	1	1	4	5	5	38.5	500	192500
FA11	1	1	1	1	4	6	6	42	500	210000
FA11	1	1	1	1	4	7	7	40.5	500	202500

experiment	block	trt	CO2	O3	Chamber	SUBREP	flower ID	AVERAGE	suspensior	TTL POLL
FA11	1	1	1	1	4	8	8	48	500	240000
FA11	1	1	1	1	4	9	9	32.25	500	161250
FA11	1	1	1	1	4	10	10	65	1000	650000
FA11	1	1	1	1	4	11	11	68	500	340000
FA11	1	4	2	2	5	1	1	69.5	1000	695000
FA11	1	4	2	2	5	2	2	91.5	500	457500
FA11	1	4	2	2	5	3	3	40	500	200000
FA11	1	4	2	2	5	4	4	28	500	140000
FA11	1	4	2	2	5	5	5	79.25	1000	792500
FA11	1	4	2	2	5	6	6	14	500	70000
FA11	1	4	2	2	5	7	7	29	1000	290000
FA11	1	4	2	2	5	8	8	110.75	1000	1107500
FA11	1	4	2	2	5	9	9			
FA11	1	4	2	2	5	10	10	48.5	500	242500
FA11	1	4	2	2	5	11	11	54.5	500	272500
FA11	1	4	2	2	5	12	12	98.75	500	493750
FA11	1	4	2	2	5	13	13	19	500	95000
FA11	1	4	2	2	5	14	14	76.5	1000	765000
FA11	1	4	2	2	5	15	15			
FA11	2	1	1	1	6	1	1	30.5	500	152500
FA11	2	1	1	1	6	2	2	34	500	170000
FA11	2	3	2	1	7	1	1	65	1000	650000
FA11	2	3	2	1	7	2	2	15	500	75000
FA11	2	3	2	1	7	3	3	69.5	500	347500
FA11	2	3	2	1	7	4	4			
FA11	2	3	2	1	7	5	5	89.5	500	447500
FA11	2	3	2	1	7	6	6			

experiment	block	trt	CO2	O3	Chamber	SUBREP	flower ID	AVERAGE	suspensior	TTL POLL
FA11	2	2	1	2	8	1				
SP11	3	2	1	2	1	1	22	0	500	0
SP11	3	2	1	2	1	2	18A	0	500	0
SP11	3	2	1	2	1	3	18B	4	500	20000
SP11	3	2	1	2	1	4	24A	13	500	65000
SP11	3	2	1	2	1	5	24B	0	500	0
SP11	3	2	1	2	1	6	24C	14	500	70000
SP11	3	2	1	2	1	7	24D	8.5	500	42500
SP11	3	2	1	2	1	8	24E	0	500	0
SP11	3	2	1	2	1	9	29A	0	500	0
SP11	3	2	1	2	1	10	29B	0	500	0
SP11	3	2	1	2	1	11	32A	12	500	60000
SP11	3	2	1	2	1	12	32B			
SP11	3	2	1	2	1	13	32C	2.5	500	12500
SP11	3	4	2	2	2	1	36	0	500	0
SP11	3	4	2	2	2	2	40	8.5	500	42500
SP11	3	4	2	2	2	3	44	0	500	0
SP11	3	4	2	2	2	4	23A	20	500	100000
SP11	3	4	2	2	2	5	23B	5	500	25000
SP11	3	4	2	2	2	6	23C	48	500	240000
SP11	3	4	2	2	2	7	29A	5	500	25000
SP11	3	4	2	2	2	8	29B	7	500	35000
SP11	3	4	2	2	2	9	2A	10	500	50000
SP11	3	4	2	2	2	10	2B	0	500	0
SP11	3	4	2	2	2	11	2C	0	500	0
SP11	3	4	2	2	2	12	38A	10	500	50000
SP11	3	4	2	2	2	13	38B	4	500	20000

experiment	block	trt	CO2	O3	Chamber	SUBREP	flower ID	AVERAGE	suspensior	TTL POLL
SP11	3	4	2	2	2	14	38C	0	500	0
SP11	3	4	2	2	2	15	43A	0	500	0
SP11	3	4	2	2	2	16	43B			
SP11	3	4	2	2	2	17	43C	4	500	20000
SP11	3	4	2	2	2	18	43D	14	500	70000
SP11	3	4	2	2	2	19	43E	0	500	0
SP11	3	4	2	2	2	20	45A	8.5	500	42500
SP11	3	4	2	2	2	21	45B	19.5	500	97500
SP11	3	4	2	2	2	22	45C	9.5	500	47500
SP11	3	1	1	1	3	1	6	2.5	500	12500
SP11	3	1	1	1	3	2	27	3	500	15000
SP11	3	1	1	1	3	3	37			
SP11	3	1	1	1	3	4	41	0	500	0
SP11	3	1	1	1	3	5	29A	0	500	0
SP11	3	1	1	1	3	6	29B	5.5	500	27500
SP11	3	1	1	1	3	7	29C	1.5	500	7500
SP11	3	1	1	1	3	8	9A	5.5	500	27500
SP11	3	1	1	1	3	9	9B	3	500	15000
SP11	3	3	2	1	4	1	16			
SP11	3	3	2	1	4	2	17	0	500	0
SP11	3	3	2	1	4	3	30	6	500	30000
SP11	3	3	2	1	4	4	31			
SP11	3	3	2	1	4	5	36	4.5	500	22500
SP11	3	3	2	1	4	6	20A	13	500	65000
SP11	3	3	2	1	4	7	20B			
SP11	3	3	2	1	4	8	24A	0	500	0
SP11	3	3	2	1	4	9	24B	0	500	0

experiment	block	trt	CO2	O3	Chamber	SUBREP	flower ID	AVERAGE	suspensior	TTL POLL
SP11	3	3	2	1	4	10	24C	2	500	10000
SP11	3	3	2	1	4	11	25A			
SP11	3	3	2	1	4	12	25B			
SP11	3	3	2	1	4	13	25C	22.5	500	112500
SP11	3	3	2	1	4	14	27A	35	500	175000
SP11	3	3	2	1	4	15	27B			
SP11	3	3	2	1	4	16	27C	22	500	110000
SP11	3	3	2	1	4	17	33A	0	500	0
SP11	3	3	2	1	4	18	33B	25.5	500	127500
SP11	3	3	2	1	4	19	33C	8	500	40000
SP11	3	3	2	1	4	20	33D	9	500	45000
SP11	3	3	2	1	4	21	33E			
SP11	3	3	2	1	4	22	9A			
SP11	3	3	2	1	4	23	9B			
SP11	3	3	2	1	4	24	9C	2.5	500	12500
SP11	3	3	2	1	4	25	9D	11.5	500	57500
SP11	4	2	1	2	5	1	1	0	500	0
SP11	4	2	1	2	5	2	19	9.5	500	47500
SP11	4	2	1	2	5	3	20	0	500	0
SP11	4	2	1	2	5	4	22			
SP11	4	2	1	2	5	5	26	8.5	500	42500
SP11	4	2	1	2	5	6	38	0	500	0
SP11	4	2	1	2	5	7	41	14	500	70000
SP11	4	2	1	2	5	8	12A	0	500	0
SP11	4	2	1	2	5	9	12B	51.5	500	257500
SP11	4	2	1	2	5	10	14A	0	500	0
SP11	4	2	1	2	5	11	14B	12	500	60000

experiment	block	trt	CO2	O3	Chamber	SUBREP	flower ID	AVERAGE	suspensior	TTL POLL
SP11	4	2	1	2	5	12	14C	4.5	500	22500
SP11	4	2	1	2	5	13	40A	0	500	0
SP11	4	2	1	2	5	14	40B	4	500	20000
SP11	4	2	1	2	5	15	40C	3.5	500	17500
SP11	4	2	1	2	5	16	40D	0	500	0
SP11	4	2	1	2	5	17	40E	0	500	0
SP11	4	3	2	1	6	1	5	14	500	70000
SP11	4	3	2	1	6	2	28	0	500	0
SP11	4	3	2	1	6	3	29			
SP11	4	3	2	1	6	4	37	0	500	0
SP11	4	3	2	1	6	5	25A	4.5	500	22500
SP11	4	3	2	1	6	6	25B			
SP11	4	3	2	1	6	7	27A	30.5	500	152500
SP11	4	3	2	1	6	8	27B	23.5	500	117500
SP11	4	3	2	1	6	9	27C	6.5	500	32500
SP11	4	3	2	1	6	10	27D			
SP11	4	3	2	1	6	11	27E			
SP11	4	3	2	1	6	12	42A			
SP11	4	3	2	1	6	13	42B	0	500	0
SP11	4	3	2	1	6	14	42C			
SP11	4	1	1	1	7	1	17	0	500	0
SP11	4	1	1	1	7	2	24	8	500	40000
SP11	4	1	1	1	7	3	27			
SP11	4	1	1	1	7	4	46	8.5	500	42500
SP11	4	1	1	1	7	5	9A			
SP11	4	1	1	1	7	6	9B			
SP11	4	4	2	2	8	1	6	14	500	70000

experiment	block	trt	CO2	O3	Chamber	SUBREP	flower ID	AVERAGE	suspensior	TTL POLL
SP11	4	4	2	2	8	2	12	8.5	500	42500
SP11	4	4	2	2	8	3	28	0	500	0
SP11	4	4	2	2	8	4	42	0	500	0
SP11	4	4	2	2	8	5	45	0	500	0
SP11	4	4	2	2	8	6	19A			
SP11	4	4	2	2	8	7	19B	0	500	0
SP11	4	4	2	2	8	8	19C	0	500	0
SP11	4	4	2	2	8	9	29A	0	500	0
SP11	4	4	2	2	8	10	29B	3	500	15000
SP11	4	4	2	2	8	11	39A			
SP11	4	4	2	2	8	12	39B	0	500	0
SP11	4	4	2	2	8	13	3A	20.5	500	102500
SP11	4	4	2	2	8	14	3B	3.5	500	17500
SP11	4	4	2	2	8	15	3C	8	500	40000
SP11	4	4	2	2	8	16	3D	44.5	500	222500
SP12	5	4	2	2	1	1	1	25	500	125000
SP12	5	4	2	2	1	2	2	11.5	500	57500
SP12	5	4	2	2	1	3	3	22	500	110000
SP12	5	4	2	2	1	4	4	13	500	65000
SP12	5	4	2	2	1	5	5	54.5	500	272500
SP12	5	1	1	1	2	1	1	27.5	500	137500
SP12	5	1	1	1	2	2	2	17.5	500	87500
SP12	5	1	1	1	2	3	3	7.5	500	500
SP12	5	1	1	1	2	4	4	138.5	500	692500
SP12	5	1	1	1	2	5	5	3.5	500	17500
SP12	5	3	2	1	3	1	1	44	500	220000
SP12	5	3	2	1	3	2	2	24	500	120000

experiment	block	trt	CO2	O3	Chamber	SUBREP	flower ID	AVERAGE	suspensior	TTL POLL
SP12	5	3	2	1	3	3	3	19	500	95000
SP12	5	3	2	1	3	4	4	25.5	500	127500
SP12	5	3	2	1	3	5	5	38	500	190000
SP12	5	3	2	1	3	6	6			
SP12	5	3	2	1	3	7	7	55.5	500	277500
SP12	5	3	2	1	3	8	8			
SP12	5	3	2	1	3	9	9	14.5	500	72500
SP12	6	1	1	1	4	1	1	14	500	70000
SP12	6	1	1	1	4	2	2	9.5	500	47500
SP12	6	1	1	1	4	3	3	12	500	60000
SP12	6	1	1	1	4	4	4	12	500	60000
SP12	6	4	2	2	5	1	1	26.5	500	132500
SP12	6	4	2	2	5	2	2	19.33333	500	96666.67
SP12	6	4	2	2	5	3	3	31	500	155000
SP12	6	4	2	2	5	4	4	8.5	500	42500
SP12	6	4	2	2	5	5	5	26.5	500	132500
SP12	5	2	1	2	6	1				
SP12	6	3	2	1	7	1	1			
SP12	6	3	2	1	7	2	2			
SP12	6	3	2	1	7	3	3	5.5	500	27500
SP12	6	3	2	1	7	4	4	65	500	325000
SP12	5	2	1	2	8	1	1	22	500	110000
SP12	5	2	1	2	8	2	2	4.5	500	22500

Phleum pratense L.

treatments

experiment	block	trt	CO2	O3	Chamber	SUBREP	flower ID	AVERAGE	suspensior	TTL POLL
	ambient (1)	elevated (2)			trt 1	trt 2	trt 3			
CO2	400 ppm	800 ppm			30 ppb O3	80 ppb O3	30 ppb O3			
O3	30 ppb	80 ppb			400 ppm CO2	400 ppm C	800 ppm C			
3 experiment	FA11	SP11	SP12							
8 chambers per experiment in a complete factorial design										
2 reps (block) per experiment										
Chamber experiment (CSTR)										
All flowers collected from each chamber										
flower bagged upon emergence and immediately removed and placed in freezer after dehiscence of pc										
Flowers washed with PBS-T (3 washes; pollen removed from each wash via centrifuge; three washes cc										
pollen suspended in known volume and counted using hemocytometer (the total amount of pollen fro										
Phl-p-5 content determined using ELISA										
ALL FLOWERS WERE COLLECTED FROM ALL CHAMBERS										
Some flowers were damaged in collection (ie pollen lost) and not used in analysis of pollen content or p										

experiment	block	trt	CO2	O3	Chamber	SUBREP	flower ID	total Pollen	ng/ml Phlp5	total allergen
FA11	2	4	2	2	1	1				
FA11	1	2	1	2	2	1	1	1857500	23570.50	141423
FA11	1	2	1	2	2	2	2	3840000	28167.00	197169
FA11	1	2	1	2	2	3	3	4001250	40737.33	285161.3
FA11	1	2	1	2	2	4	4	3060000	8489.75	59428.25
FA11	1	3	2	1	3	1	1	1560000	17722.33	124056.3
FA11	1	3	2	1	3	2	2	4691250	25817.67	180723.7
FA11	1	3	2	1	3	3	3	3958750	19371.00	135597
FA11	1	3	2	1	3	4	4			
FA11	1	3	2	1	3	5	5			
FA11	1	3	2	1	3	6	6			
FA11	1	3	2	1	3	7	7			
FA11	1	3	2	1	3	8	8	4537500	52992.00	370944
FA11	1	3	2	1	3	9	9	2226250	23173.67	162215.7
FA11	1	3	2	1	3	10	10	3747500	49917.00	349419
FA11	1	3	2	1	3	11	11	1087500	6453.50	38721
FA11	1	3	2	1	3	12	12	2083750	12797.00	76782
FA11	1	3	2	1	3	13	13	2698750	28378.67	198650.7
FA11	1	3	2	1	3	14	14	4332500	26120.67	182844.7
FA11	1	3	2	1	3	15	15	3347500	34654.33	242580.3
FA11	1	1	1	1	4	1	1	1126250	16539.33	115775.3
FA11	1	1	1	1	4	2	2	1296250	13259.75	92818.25
FA11	1	1	1	1	4	3	3	1685000	12248.67	85740.67
FA11	1	1	1	1	4	4	4	2135000	14595.75	102170.3
FA11	1	1	1	1	4	5	5	1010000	2680.67	18764.67
FA11	1	1	1	1	4	6	6	1895000	14389.33	100725.3
FA11	1	1	1	1	4	7	7	1018750	5728.75	40101.25

experiment	block	trt	CO2	O3	Chamber	SUBREP	flower ID	total Pollen	ng/ml Phlp5	total allergen
FA11	1	1	1	1	4	8	8	2137500	27336.67	191356.7
FA11	1	1	1	1	4	9	9	1237500	4796.33	33574.33
FA11	1	1	1	1	4	10	10	4036250	32439.67	227077.7
FA11	1	1	1	1	4	11	11	1967500	16215.50	113508.5
FA11	1	4	2	2	5	1	1	5206250	8841.00	61887
FA11	1	4	2	2	5	2	2	5095000	21369.00	149583
FA11	1	4	2	2	5	3	3	2242500	32982.50	230877.5
FA11	1	4	2	2	5	4	4	1171667	3321.75	23252.25
FA11	1	4	2	2	5	5	5	3605000	58306.50	408145.5
FA11	1	4	2	2	5	6	6	1102500	6328.00	44296
FA11	1	4	2	2	5	7	7	3473750	14776.00	103432
FA11	1	4	2	2	5	8	8	6060000	32498.00	227486
FA11	1	4	2	2	5	9	9			
FA11	1	4	2	2	5	10	10	3578750	45729.00	320103
FA11	1	4	2	2	5	11	11	2765000	15944.50	95667
FA11	1	4	2	2	5	12	12	3497500	6837.25	47860.75
FA11	1	4	2	2	5	13	13	1965000	2899.00	17394
FA11	1	4	2	2	5	14	14	4000000	27108.00	189756
FA11	1	4	2	2	5	15	15			
FA11	2	1	1	1	6	1	1	1712500	11489.67	80427.67
FA11	2	1	1	1	6	2	2	1497500	14367.00	86202
FA11	2	3	2	1	7	1	1	4110000	36303.67	254125.7
FA11	2	3	2	1	7	2	2	1582500	5609.00	33654
FA11	2	3	2	1	7	3	3	3492500	23657.33	165601.3
FA11	2	3	2	1	7	4	4			
FA11	2	3	2	1	7	5	5	5080000	51044.50	357311.5
FA11	2	3	2	1	7	6	6			

experiment	block	trt	CO2	O3	Chamber	SUBREP	flower ID	total Pollen	ng/ml Phlp5	total allergen
FA11	2	2	1	2	8	1				
SP11	3	2	1	2	1	1	22	255000	1283.67	3851
SP11	3	2	1	2	1	2	18A	248333.3	1394.00	4182
SP11	3	2	1	2	1	3	18B	1140000	1174.00	7044
SP11	3	2	1	2	1	4	24A	1272500	2141.00	12846
SP11	3	2	1	2	1	5	24B	667916.7	539.50	1618.5
SP11	3	2	1	2	1	6	24C	1342500	762.67	4576
SP11	3	2	1	2	1	7	24D	1557500	2770.67	16624
SP11	3	2	1	2	1	8	24E	271666.7	974.50	2923.5
SP11	3	2	1	2	1	9	29A	923333.3	931.67	2795
SP11	3	2	1	2	1	10	29B	220000	1122.67	3368
SP11	3	2	1	2	1	11	32A	1125000	299.75	1798.5
SP11	3	2	1	2	1	12	32B			
SP11	3	2	1	2	1	13	32C	555000	770.00	4620
SP11	3	4	2	2	2	1	36	1241667	13021.00	39063
SP11	3	4	2	2	2	2	40	632500	1241.00	7446
SP11	3	4	2	2	2	3	44	461666.7	2974.00	8922
SP11	3	4	2	2	2	4	23A	1885000	2321.67	13930
SP11	3	4	2	2	2	5	23B	697500	516.33	3098
SP11	3	4	2	2	2	6	23C	1015000	2011.00	12066
SP11	3	4	2	2	2	7	29A	770000	19954.33	119726
SP11	3	4	2	2	2	8	29B	862500	4000.50	24003
SP11	3	4	2	2	2	9	2A	595000	3129.67	18778
SP11	3	4	2	2	2	10	2B	1880000	557.00	1671
SP11	3	4	2	2	2	11	2C	451250	10318.67	30956
SP11	3	4	2	2	2	12	38A	1355000	587.67	3526
SP11	3	4	2	2	2	13	38B	745000	2105.00	12630

experiment	block	trt	CO2	O3	Chamber	SUBREP	flower ID	total Pollen	ng/ml Phlp5	total allergen
SP11	3	4	2	2	2	14	38C	518333.3	3684.33	11053
SP11	3	4	2	2	2	15	43A	205000	4700.50	14101.5
SP11	3	4	2	2	2	16	43B			
SP11	3	4	2	2	2	17	43C	1430000	770.00	4620
SP11	3	4	2	2	2	18	43D	1437500	1220.00	7320
SP11	3	4	2	2	2	19	43E	705000	2078.00	6234
SP11	3	4	2	2	2	20	45A	900000	1736.00	10416
SP11	3	4	2	2	2	21	45B	1292500	2407.67	14446
SP11	3	4	2	2	2	22	45C	1007500	12948.00	77688
SP11	3	1	1	1	3	1	6	500000	2410.33	14462
SP11	3	1	1	1	3	2	27	722500	535.67	3214
SP11	3	1	1	1	3	3	37			
SP11	3	1	1	1	3	4	41	470000	888.75	2666.25
SP11	3	1	1	1	3	5	29A	705000	933.67	2801
SP11	3	1	1	1	3	6	29B	777500	537.67	3226
SP11	3	1	1	1	3	7	29C	495000	1293.33	7760
SP11	3	1	1	1	3	8	9A	985000	813.67	4882
SP11	3	1	1	1	3	9	9B	820000	2703.00	16218
SP11	3	3	2	1	4	1	16			
SP11	3	3	2	1	4	2	17	893750	525.50	2102
SP11	3	3	2	1	4	3	30	600000	2977.50	17865
SP11	3	3	2	1	4	4	31			
SP11	3	3	2	1	4	5	36	842500	2970.33	17822
SP11	3	3	2	1	4	6	20A	990000	1607.00	9642
SP11	3	3	2	1	4	7	20B			
SP11	3	3	2	1	4	8	24A	1453333	1128.25	3384.75
SP11	3	3	2	1	4	9	24B	81666.67	1913.75	5741.25

experiment	block	trt	CO2	O3	Chamber	SUBREP	flower ID	total Pollen	ng/ml Phlp5	total allergen
SP11	3	3	2	1	4	10	24C	762500	2096.67	12580
SP11	3	3	2	1	4	11	25A			
SP11	3	3	2	1	4	12	25B			
SP11	3	3	2	1	4	13	25C	1750000	2680.33	16082
SP11	3	3	2	1	4	14	27A	1512500	37031.00	222186
SP11	3	3	2	1	4	15	27B			
SP11	3	3	2	1	4	16	27C	2090000	4688.00	28128
SP11	3	3	2	1	4	17	33A	868333.3	646.75	1940.25
SP11	3	3	2	1	4	18	33B	2092500	2999.00	17994
SP11	3	3	2	1	4	19	33C	1130000	1658.75	9952.5
SP11	3	3	2	1	4	20	33D	1082500	2661.67	15970
SP11	3	3	2	1	4	21	33E			
SP11	3	3	2	1	4	22	9A			
SP11	3	3	2	1	4	23	9B			
SP11	3	3	2	1	4	24	9C	727500	818.25	4909.5
SP11	3	3	2	1	4	25	9D	1817500	1578.25	9469.5
SP11	4	2	1	2	5	1	1	1037500	4204.67	8409.333
SP11	4	2	1	2	5	2	19	522500	1982.00	11892
SP11	4	2	1	2	5	3	20	215000	894.00	1788
SP11	4	2	1	2	5	4	22			
SP11	4	2	1	2	5	5	26	252500	1189.75	7138.5
SP11	4	2	1	2	5	6	38	956666.7	1284.25	2568.5
SP11	4	2	1	2	5	7	41	1880000	2751.33	16508
SP11	4	2	1	2	5	8	12A	730000	3455.67	6911.333
SP11	4	2	1	2	5	9	12B	962500	1473.00	8838
SP11	4	2	1	2	5	10	14A	1110000	5634.00	11268
SP11	4	2	1	2	5	11	14B	1202500	14661.67	87970

experiment	block	trt	CO2	O3	Chamber	SUBREP	flower ID	total Pollen	ng/ml Phlp5	total allergen
SP11	4	2	1	2	5	12	14C	830000	3876.67	23260
SP11	4	2	1	2	5	13	40A	731666.7	4221.00	8442
SP11	4	2	1	2	5	14	40B	960000	3165.67	18994
SP11	4	2	1	2	5	15	40C	725000	1935.00	11610
SP11	4	2	1	2	5	16	40D	1127500	9558.50	19117
SP11	4	2	1	2	5	17	40E	216666.7	1427.00	2854
SP11	4	3	2	1	6	1	5	1002500	418.25	2509.5
SP11	4	3	2	1	6	2	28	638333.3	4258.00	12774
SP11	4	3	2	1	6	3	29			
SP11	4	3	2	1	6	4	37	423333.3	1255.25	3765.75
SP11	4	3	2	1	6	5	25A	960000	1331.25	7987.5
SP11	4	3	2	1	6	6	25B			
SP11	4	3	2	1	6	7	27A	2360000	3422.50	20535
SP11	4	3	2	1	6	8	27B	2335000	4685.50	28113
SP11	4	3	2	1	6	9	27C	1247500	3223.33	19340
SP11	4	3	2	1	6	10	27D			
SP11	4	3	2	1	6	11	27E			
SP11	4	3	2	1	6	12	42A			
SP11	4	3	2	1	6	13	42B	379583.3	2193.50	6580.5
SP11	4	3	2	1	6	14	42C			
SP11	4	1	1	1	7	1	17	186666.7	449.67	1798.667
SP11	4	1	1	1	7	2	24	1067500	5240.00	31440
SP11	4	1	1	1	7	3	27			
SP11	4	1	1	1	7	4	46	937500	2924.67	17548
SP11	4	1	1	1	7	5	9A			
SP11	4	1	1	1	7	6	9B			
SP11	4	4	2	2	8	1	6	1542500	1664.33	9986

experiment	block	trt	CO2	O3	Chamber	SUBREP	flower ID	total Pollen	ng/ml Phlp5	total allergen
SP11	4	4	2	2	8	2	12	712500	1711.33	10268
SP11	4	4	2	2	8	3	28	707500	1826.00	5478
SP11	4	4	2	2	8	4	42	1765000	9462.00	28386
SP11	4	4	2	2	8	5	45	1010833	1116.00	3348
SP11	4	4	2	2	8	6	19A			
SP11	4	4	2	2	8	7	19B	350000	2532.00	7596
SP11	4	4	2	2	8	8	19C	410000	373.00	1119
SP11	4	4	2	2	8	9	29A	198333.3	128.33	385
SP11	4	4	2	2	8	10	29B	917500	1508.25	9049.5
SP11	4	4	2	2	8	11	39A			
SP11	4	4	2	2	8	12	39B	451666.7	775.00	2325
SP11	4	4	2	2	8	13	3A	2142500	2323.33	13940
SP11	4	4	2	2	8	14	3B	557500	322.25	1933.5
SP11	4	4	2	2	8	15	3C	830000	552.00	3312
SP11	4	4	2	2	8	16	3D	2305000	8483.00	50898
SP12	5	4	2	2	1	1	1	996250	8512.00	51072
SP12	5	4	2	2	1	2	2	1037500	5707.25	34243.5
SP12	5	4	2	2	1	3	3	2201250	7967.67	47806
SP12	5	4	2	2	1	4	4	1450000	8996.00	53976
SP12	5	4	2	2	1	5	5	2150000	12993.75	90956.25
SP12	5	1	1	1	2	1	1	1291250	4739.50	28437
SP12	5	1	1	1	2	2	2	1297500	11566.67	69400
SP12	5	1	1	1	2	3	3	650500	7943.33	47660
SP12	5	1	1	1	2	4	4	1245000	43550.50	261303
SP12	5	1	1	1	2	5	5	350000	5191.00	31146
SP12	5	3	2	1	3	1	1	2706250	28058.00	168348
SP12	5	3	2	1	3	2	2	792500	37611.00	225666

experiment	block	trt	CO2	O3	Chamber	SUBREP	flower ID	total Pollen	ng/ml Phlp5	total allergen
SP12	5	3	2	1	3	3	3	933750	6767.50	40605
SP12	5	3	2	1	3	4	4	1817500	27700.00	166200
SP12	5	3	2	1	3	5	5	1002500	9470.00	66290
SP12	5	3	2	1	3	6	6			
SP12	5	3	2	1	3	7	7	1686250	34223.00	205338
SP12	5	3	2	1	3	8	8			
SP12	5	3	2	1	3	9	9	1987500	11350.25	68101.5
SP12	6	1	1	1	4	1	1	2311250	28713.00	172278
SP12	6	1	1	1	4	2	2	828750	3142.67	18856
SP12	6	1	1	1	4	3	3	1537500	5299.75	37098.25
SP12	6	1	1	1	4	4	4	975000	4136.00	24816
SP12	6	4	2	2	5	1	1	1398750	15684.00	94104
SP12	6	4	2	2	5	2	2	2460417	17281.50	103689
SP12	6	4	2	2	5	3	3	2190000	13467.67	80806
SP12	6	4	2	2	5	4	4	1135000	10601.33	63608
SP12	6	4	2	2	5	5	5	1840000	7771.25	46627.5
SP12	5	2	1	2	6	1				
SP12	6	3	2	1	7	1	1			
SP12	6	3	2	1	7	2	2			
SP12	6	3	2	1	7	3	3	397500	3225.33	19352
SP12	6	3	2	1	7	4	4	3242500	19247.00	134729
SP12	5	2	1	2	8	1	1	472500	156.25	1093.75
SP12	5	2	1	2	8	2	2	307500	84.75	508.5

Phleum pratense L.

treatments

experiment	block	trt	CO2	O3	Chamber	SUBREP	flower ID	total Pollen	ng/ml Phlp5	total allergen
	ambient (1)	elevated (2)			trt 1	trt 2	trt 3			
CO2	400 ppm	800 ppm			30 ppb O3	80 ppb O3	30 ppb O3			
O3	30 ppb	80 ppb			400 ppm CO2	400 ppm C	800 ppm C			
3 experiment	FA11	SP11	SP12							
8 chambers per experiment in a complete factorial design										
2 reps (block) per experiment										
Chamber experiment (CSTR)										
All flowers collected from each chamber										
flower bagged upon emergence and immediately removed and placed in freezer after dehiscence of po										
Flowers washed with PBS-T (3 washes; pollen removed from each wash via centrifuge; three washes co										
pollen suspended in known volume and counted using hemocytometer (the total amount of pollen fro										
Phl-p-5 content determined using ELISA										
ALL FLOWERS WERE COLLECTED FROM ALL CHAMBERS										
Some flowers were damaged in collection (ie pollen lost) and not used in analysis of pollen content or p										

experiment	block	trt	CO2	O3	Chamber	SUBREP	flower ID	Total ML PBS-T wash	allergen per pollen grain
FA11	2	4	2	2	1	1			
FA11	1	2	1	2	2	1	1	6	0.0761362
FA11	1	2	1	2	2	2	2	7	0.05134609
FA11	1	2	1	2	2	3	3	7	0.07126806
FA11	1	2	1	2	2	4	4	7	0.019421
FA11	1	3	2	1	3	1	1	7	0.07952329
FA11	1	3	2	1	3	2	2	7	0.03852356
FA11	1	3	2	1	3	3	3	7	0.03425248
FA11	1	3	2	1	3	4	4		
FA11	1	3	2	1	3	5	5		
FA11	1	3	2	1	3	6	6		
FA11	1	3	2	1	3	7	7		
FA11	1	3	2	1	3	8	8	7	0.08175074
FA11	1	3	2	1	3	9	9	7	0.07286498
FA11	1	3	2	1	3	10	10	7	0.09324056
FA11	1	3	2	1	3	11	11	6	0.03560552
FA11	1	3	2	1	3	12	12	6	0.03684799
FA11	1	3	2	1	3	13	13	7	0.0736084
FA11	1	3	2	1	3	14	14	7	0.04220304
FA11	1	3	2	1	3	15	15	7	0.07246612
FA11	1	1	1	1	4	1	1	7	0.10279719
FA11	1	1	1	1	4	2	2	7	0.07160521
FA11	1	1	1	1	4	3	3	7	0.05088467
FA11	1	1	1	1	4	4	4	7	0.04785492
FA11	1	1	1	1	4	5	5	7	0.01857888
FA11	1	1	1	1	4	6	6	7	0.05315321
FA11	1	1	1	1	4	7	7	7	0.03936319

experiment	block	trt	CO2	O3	Chamber	SUBREP	flower ID	Total ML PBS-T wash	allergen per pollen grain
FA11	1	1	1	1	4	8	8	7	0.08952359
FA11	1	1	1	1	4	9	9	7	0.02713077
FA11	1	1	1	1	4	10	10	7	0.05625956
FA11	1	1	1	1	4	11	11	7	0.05769174
FA11	1	4	2	2	5	1	1	7	0.01188706
FA11	1	4	2	2	5	2	2	7	0.02935878
FA11	1	4	2	2	5	3	3	7	0.10295541
FA11	1	4	2	2	5	4	4	7	0.01984545
FA11	1	4	2	2	5	5	5	7	0.1132165
FA11	1	4	2	2	5	6	6	7	0.04017778
FA11	1	4	2	2	5	7	7	7	0.02977531
FA11	1	4	2	2	5	8	8	7	0.03753894
FA11	1	4	2	2	5	9	9		
FA11	1	4	2	2	5	10	10	7	0.08944548
FA11	1	4	2	2	5	11	11	6	0.03459928
FA11	1	4	2	2	5	12	12	7	0.01368427
FA11	1	4	2	2	5	13	13	6	0.00885191
FA11	1	4	2	2	5	14	14	7	0.047439
FA11	1	4	2	2	5	15	15		
FA11	2	1	1	1	6	1	1	7	0.04696506
FA11	2	1	1	1	6	2	2	6	0.05756394
FA11	2	3	2	1	7	1	1	7	0.06183106
FA11	2	3	2	1	7	2	2	6	0.02126635
FA11	2	3	2	1	7	3	3	7	0.04741627
FA11	2	3	2	1	7	4	4		
FA11	2	3	2	1	7	5	5	7	0.07033691
FA11	2	3	2	1	7	6	6		

experiment	block	trt	CO2	O3	Chamber	SUBREP	flower ID	Total ML PBS-T wash	allergen per pollen grain
FA11	2	2	1	2	8	1			
SP11	3	2	1	2	1	1	22	3	0.01510196
SP11	3	2	1	2	1	2	18A	3	0.01684027
SP11	3	2	1	2	1	3	18B	6	0.00617895
SP11	3	2	1	2	1	4	24A	6	0.01009509
SP11	3	2	1	2	1	5	24B	3	0.00242321
SP11	3	2	1	2	1	6	24C	6	0.00340857
SP11	3	2	1	2	1	7	24D	6	0.01067352
SP11	3	2	1	2	1	8	24E	3	0.01076135
SP11	3	2	1	2	1	9	29A	3	0.00302708
SP11	3	2	1	2	1	10	29B	3	0.01530909
SP11	3	2	1	2	1	11	32A	6	0.00159867
SP11	3	2	1	2	1	12	32B		
SP11	3	2	1	2	1	13	32C	6	0.00832432
SP11	3	4	2	2	2	1	36	3	0.03146013
SP11	3	4	2	2	2	2	40	6	0.01177233
SP11	3	4	2	2	2	3	44	3	0.01932563
SP11	3	4	2	2	2	4	23A	6	0.00738992
SP11	3	4	2	2	2	5	23B	6	0.00444158
SP11	3	4	2	2	2	6	23C	6	0.01188768
SP11	3	4	2	2	2	7	29A	6	0.15548831
SP11	3	4	2	2	2	8	29B	6	0.02782957
SP11	3	4	2	2	2	9	2A	6	0.03155966
SP11	3	4	2	2	2	10	2B	3	0.00088883
SP11	3	4	2	2	2	11	2C	3	0.06860055
SP11	3	4	2	2	2	12	38A	6	0.00260221
SP11	3	4	2	2	2	13	38B	6	0.01695302

experiment	block	trt	CO2	O3	Chamber	SUBREP	flower ID	Total ML PBS-T wash	allergen per pollen grain
SP11	3	4	2	2	2	14	38C	3	0.02132412
SP11	3	4	2	2	2	15	43A	3	0.0687878
SP11	3	4	2	2	2	16	43B		
SP11	3	4	2	2	2	17	43C	6	0.00323077
SP11	3	4	2	2	2	18	43D	6	0.00509217
SP11	3	4	2	2	2	19	43E	3	0.00884255
SP11	3	4	2	2	2	20	45A	6	0.01157333
SP11	3	4	2	2	2	21	45B	6	0.01117679
SP11	3	4	2	2	2	22	45C	6	0.07710968
SP11	3	1	1	1	3	1	6	6	0.028924
SP11	3	1	1	1	3	2	27	6	0.00444844
SP11	3	1	1	1	3	3	37		
SP11	3	1	1	1	3	4	41	3	0.00567287
SP11	3	1	1	1	3	5	29A	3	0.00397305
SP11	3	1	1	1	3	6	29B	6	0.0041492
SP11	3	1	1	1	3	7	29C	6	0.01567677
SP11	3	1	1	1	3	8	9A	6	0.00495635
SP11	3	1	1	1	3	9	9B	6	0.01977805
SP11	3	3	2	1	4	1	16		
SP11	3	3	2	1	4	2	17	4	0.00235189
SP11	3	3	2	1	4	3	30	6	0.029775
SP11	3	3	2	1	4	4	31		
SP11	3	3	2	1	4	5	36	6	0.02115371
SP11	3	3	2	1	4	6	20A	6	0.00973939
SP11	3	3	2	1	4	7	20B		
SP11	3	3	2	1	4	8	24A	3	0.00232896
SP11	3	3	2	1	4	9	24B	3	0.07030102

experiment	block	trt	CO2	O3	Chamber	SUBREP	flower ID	Total ML PBS-T wash	allergen per pollen grain
SP11	3	3	2	1	4	10	24C	6	0.01649836
SP11	3	3	2	1	4	11	25A		
SP11	3	3	2	1	4	12	25B		
SP11	3	3	2	1	4	13	25C	6	0.00918971
SP11	3	3	2	1	4	14	27A	6	0.14689983
SP11	3	3	2	1	4	15	27B		
SP11	3	3	2	1	4	16	27C	6	0.01345837
SP11	3	3	2	1	4	17	33A	3	0.00223445
SP11	3	3	2	1	4	18	33B	6	0.00859928
SP11	3	3	2	1	4	19	33C	6	0.00880752
SP11	3	3	2	1	4	20	33D	6	0.01475289
SP11	3	3	2	1	4	21	33E		
SP11	3	3	2	1	4	22	9A		
SP11	3	3	2	1	4	23	9B		
SP11	3	3	2	1	4	24	9C	6	0.00674845
SP11	3	3	2	1	4	25	9D	6	0.00521018
SP11	4	2	1	2	5	1	1	2	0.00810538
SP11	4	2	1	2	5	2	19	6	0.02275981
SP11	4	2	1	2	5	3	20	2	0.00831628
SP11	4	2	1	2	5	4	22		
SP11	4	2	1	2	5	5	26	6	0.02827129
SP11	4	2	1	2	5	6	38	2	0.00268484
SP11	4	2	1	2	5	7	41	6	0.00878085
SP11	4	2	1	2	5	8	12A	2	0.00946758
SP11	4	2	1	2	5	9	12B	6	0.00918234
SP11	4	2	1	2	5	10	14A	2	0.01015135
SP11	4	2	1	2	5	11	14B	6	0.07315593

experiment	block	trt	CO2	O3	Chamber	SUBREP	flower ID	Total ML PBS-T wash	allergen per pollen grain
SP11	4	2	1	2	5	12	14C	6	0.0280241
SP11	4	2	1	2	5	13	40A	2	0.01153804
SP11	4	2	1	2	5	14	40B	6	0.01978542
SP11	4	2	1	2	5	15	40C	6	0.01601379
SP11	4	2	1	2	5	16	40D	2	0.01695521
SP11	4	2	1	2	5	17	40E	2	0.01317231
SP11	4	3	2	1	6	1	5	6	0.00250324
SP11	4	3	2	1	6	2	28	3	0.02001149
SP11	4	3	2	1	6	3	29		
SP11	4	3	2	1	6	4	37	3	0.00889547
SP11	4	3	2	1	6	5	25A	6	0.00832031
SP11	4	3	2	1	6	6	25B		
SP11	4	3	2	1	6	7	27A	6	0.00870127
SP11	4	3	2	1	6	8	27B	6	0.01203983
SP11	4	3	2	1	6	9	27C	6	0.01550301
SP11	4	3	2	1	6	10	27D		
SP11	4	3	2	1	6	11	27E		
SP11	4	3	2	1	6	12	42A		
SP11	4	3	2	1	6	13	42B	3	0.01733611
SP11	4	3	2	1	6	14	42C		
SP11	4	1	1	1	7	1	17	4	0.00963571
SP11	4	1	1	1	7	2	24	6	0.02945199
SP11	4	1	1	1	7	3	27		
SP11	4	1	1	1	7	4	46	6	0.01871787
SP11	4	1	1	1	7	5	9A		
SP11	4	1	1	1	7	6	9B		
SP11	4	4	2	2	8	1	6	6	0.00647391

experiment	block	trt	CO2	O3	Chamber	SUBREP	flower ID	Total ML PBS-T wash	allergen per pollen grain
SP11	4	4	2	2	8	2	12	6	0.01441123
SP11	4	4	2	2	8	3	28	3	0.00774276
SP11	4	4	2	2	8	4	42	3	0.01608272
SP11	4	4	2	2	8	5	45	3	0.00331212
SP11	4	4	2	2	8	6	19A		
SP11	4	4	2	2	8	7	19B	3	0.02170286
SP11	4	4	2	2	8	8	19C	3	0.00272927
SP11	4	4	2	2	8	9	29A	3	0.00194118
SP11	4	4	2	2	8	10	29B	6	0.00986322
SP11	4	4	2	2	8	11	39A		
SP11	4	4	2	2	8	12	39B	3	0.0051476
SP11	4	4	2	2	8	13	3A	6	0.00650642
SP11	4	4	2	2	8	14	3B	6	0.00346816
SP11	4	4	2	2	8	15	3C	6	0.00399036
SP11	4	4	2	2	8	16	3D	6	0.02208156
SP12	5	4	2	2	1	1	1	6	0.05126424
SP12	5	4	2	2	1	2	2	6	0.03300578
SP12	5	4	2	2	1	3	3	6	0.02171766
SP12	5	4	2	2	1	4	4	6	0.03722483
SP12	5	4	2	2	1	5	5	7	0.04230523
SP12	5	1	1	1	2	1	1	6	0.02202285
SP12	5	1	1	1	2	2	2	6	0.05348748
SP12	5	1	1	1	2	3	3	6	0.07326672
SP12	5	1	1	1	2	4	4	6	0.20988193
SP12	5	1	1	1	2	5	5	6	0.08898857
SP12	5	3	2	1	3	1	1	6	0.06220711
SP12	5	3	2	1	3	2	2	6	0.28475205

experiment	block	trt	CO2	O3	Chamber	SUBREP	flower ID	Total ML PBS-T wash	allergen per pollen grain
SP12	5	3	2	1	3	3	3	6	0.04348594
SP12	5	3	2	1	3	4	4	6	0.09144429
SP12	5	3	2	1	3	5	5	7	0.06612469
SP12	5	3	2	1	3	6	6		
SP12	5	3	2	1	3	7	7	6	0.12177198
SP12	5	3	2	1	3	8	8		
SP12	5	3	2	1	3	9	9	6	0.03426491
SP12	6	1	1	1	4	1	1	6	0.07453889
SP12	6	1	1	1	4	2	2	6	0.02275234
SP12	6	1	1	1	4	3	3	7	0.02412894
SP12	6	1	1	1	4	4	4	6	0.02545231
SP12	6	4	2	2	5	1	1	6	0.06727721
SP12	6	4	2	2	5	2	2	6	0.04214286
SP12	6	4	2	2	5	3	3	6	0.03689772
SP12	6	4	2	2	5	4	4	6	0.05604229
SP12	6	4	2	2	5	5	5	6	0.02534103
SP12	5	2	1	2	6	1			
SP12	6	3	2	1	7	1	1		
SP12	6	3	2	1	7	2	2		
SP12	6	3	2	1	7	3	3	6	0.04868428
SP12	6	3	2	1	7	4	4	7	0.04155096
SP12	5	2	1	2	8	1	1	7	0.00231481
SP12	5	2	1	2	8	2	2	6	0.00165366
Phleum pratense L.									
treatments									

								Total ML PBS-T wash	allergen per pollen grain
experiment	block	trt	CO2	O3	Chamber	SUBREP	flower ID		
	ambient (1)	elevated (2)			trt 1	trt 2	trt 3		
CO2	400 ppm	800 ppm			30 ppb O3	80 ppb O3	30 ppb O3		
O3	30 ppb	80 ppb			400 ppm CO2	400 ppm C	800 ppm C		
3 experiment	FA11	SP11	SP12						
8 chambers per experiment in a complete factorial design									
2 reps (block) per experiment									
Chamber experiment (CSTR)									
All flowers collected from each chamber									
flower bagged upon emergence and immediately removed and placed in freezer after dehiscence of pc									
Flowers washed with PBS-T (3 washes; pollen removed from each wash via centrifuge; three washes cc									
pollen suspended in known volume and counted using hemocytometer (the total amount of pollen fro									
Phl-p-5 content determined using ELISA									
ALL FLOWERS WERE COLLECTED FROM ALL CHAMBERS									
Some flowers were damaged in collection (ie pollen lost) and not used in analysis of pollen content or p									

experiment	block	trt	CO2	O3	Chamber	SUBREP	flower ID	flower length	flower wt
FA11	2	4	2	2	1	1			
FA11	1	2	1	2	2	1	1	5.7	0.0832
FA11	1	2	1	2	2	2	2	8.2	0.2002
FA11	1	2	1	2	2	3	3	8.2	0.2013
FA11	1	2	1	2	2	4	4	8.6	0.2219
FA11	1	3	2	1	3	1	1	6.1	0.1476
FA11	1	3	2	1	3	2	2	7.6	0.2246
FA11	1	3	2	1	3	3	3	9.8	0.202
FA11	1	3	2	1	3	4	4		
FA11	1	3	2	1	3	5	5		
FA11	1	3	2	1	3	6	6		
FA11	1	3	2	1	3	7	7		
FA11	1	3	2	1	3	8	8	9	0.2848
FA11	1	3	2	1	3	9	9	8	0.1454
FA11	1	3	2	1	3	10	10	9.2	0.3523
FA11	1	3	2	1	3	11	11	4	0.0843
FA11	1	3	2	1	3	12	12	3.4	0.0974
FA11	1	3	2	1	3	13	13	7.8	0.2592
FA11	1	3	2	1	3	14	14	9.7	0.2728
FA11	1	3	2	1	3	15	15	9.7	0.3095
FA11	1	1	1	1	4	1	1	7.9	0.3586
FA11	1	1	1	1	4	2	2	8.8	0.3018
FA11	1	1	1	1	4	3	3	12.2	0.4363
FA11	1	1	1	1	4	4	4	11.4	0.3879
FA11	1	1	1	1	4	5	5	11	0.4025
FA11	1	1	1	1	4	6	6	11.3	0.371
FA11	1	1	1	1	4	7	7	7.4	0.3445

experiment	block	trt	CO2	O3	Chamber	SUBREP	flower ID	flower length	flower wt
FA11	1	1	1	1	4	8	8	7.2	0.3048
FA11	1	1	1	1	4	9	9	10.4	0.4052
FA11	1	1	1	1	4	10	10	11.4	0.3503
FA11	1	1	1	1	4	11	11	6.2	0.1754
FA11	1	4	2	2	5	1	1	13	0.3412
FA11	1	4	2	2	5	2	2	9.2	0.3079
FA11	1	4	2	2	5	3	3	9.2	0.3123
FA11	1	4	2	2	5	4	4	9.6	0.3586
FA11	1	4	2	2	5	5	5	11.1	0.4317
FA11	1	4	2	2	5	6	6	5.2	0.123
FA11	1	4	2	2	5	7	7	9.7	0.2312
FA11	1	4	2	2	5	8	8	12	0.4488
FA11	1	4	2	2	5	9	9		
FA11	1	4	2	2	5	10	10	10.1	0.331
FA11	1	4	2	2	5	11	11	4.4	0.1182
FA11	1	4	2	2	5	12	12	6.7	0.2195
FA11	1	4	2	2	5	13	13	4.6	0.081
FA11	1	4	2	2	5	14	14	9.3	0.3006
FA11	1	4	2	2	5	15	15		
FA11	2	1	1	1	6	1	1	6.7	0.1288
FA11	2	1	1	1	6	2	2	5.6	0.1267
FA11	2	3	2	1	7	1	1	9.5	0.4452
FA11	2	3	2	1	7	2	2	3	0.086
FA11	2	3	2	1	7	3	3	7.4	0.1912
FA11	2	3	2	1	7	4	4		
FA11	2	3	2	1	7	5	5	10	0.4563
FA11	2	3	2	1	7	6	6		

experiment	block	trt	CO2	O3	Chamber	SUBREP	flower ID	flower length	flower wt
FA11	2	2	1	2	8	1			
SP11	3	2	1	2	1	1	22	1.9	0.0217
SP11	3	2	1	2	1	2	18A	2.2	0.0212
SP11	3	2	1	2	1	3	18B	3.3	0.0356
SP11	3	2	1	2	1	4	24A	4.5	0.0606
SP11	3	2	1	2	1	5	24B	4.5	0.0472
SP11	3	2	1	2	1	6	24C	4.1	0.0654
SP11	3	2	1	2	1	7	24D	2.5	0.0487
SP11	3	2	1	2	1	8	24E	3	0.0455
SP11	3	2	1	2	1	9	29A	3.5	0.0484
SP11	3	2	1	2	1	10	29B	2.1	0.0301
SP11	3	2	1	2	1	11	32A	3.65	0.0568
SP11	3	2	1	2	1	12	32B		
SP11	3	2	1	2	1	13	32C	1.7	0.0185
SP11	3	4	2	2	2	1	36	5	0.1174
SP11	3	4	2	2	2	2	40	4.9	0.1287
SP11	3	4	2	2	2	3	44	2.2	0.0462
SP11	3	4	2	2	2	4	23A	3.6	0.0763
SP11	3	4	2	2	2	5	23B	1	0.0214
SP11	3	4	2	2	2	6	23C	3.4	0.0695
SP11	3	4	2	2	2	7	29A	2.9	0.036
SP11	3	4	2	2	2	8	29B	3.2	0.045
SP11	3	4	2	2	2	9	2A	5	0.0993
SP11	3	4	2	2	2	10	2B	4.5	0.0808
SP11	3	4	2	2	2	11	2C	3.3	0.0602
SP11	3	4	2	2	2	12	38A	2.3	0.0471
SP11	3	4	2	2	2	13	38B	2.4	0.0462

experiment	block	trt	CO2	O3	Chamber	SUBREP	flower ID	flower length	flower wt
SP11	3	4	2	2	2	14	38C	1.7	0.0301
SP11	3	4	2	2	2	15	43A	4.5	0.0653
SP11	3	4	2	2	2	16	43B		
SP11	3	4	2	2	2	17	43C	2.8	0.0481
SP11	3	4	2	2	2	18	43D	3.1	0.0517
SP11	3	4	2	2	2	19	43E	3.2	0.0658
SP11	3	4	2	2	2	20	45A	2.6	0.0466
SP11	3	4	2	2	2	21	45B	2.8	0.0626
SP11	3	4	2	2	2	22	45C	3	0.0662
SP11	3	1	1	1	3	1	6	3.4	0.0529
SP11	3	1	1	1	3	2	27	2	0.0288
SP11	3	1	1	1	3	3	37		
SP11	3	1	1	1	3	4	41	3.5	0.0704
SP11	3	1	1	1	3	5	29A	2.9	0.0398
SP11	3	1	1	1	3	6	29B	2.5	0.0301
SP11	3	1	1	1	3	7	29C	3	0.051
SP11	3	1	1	1	3	8	9A	3	0.0401
SP11	3	1	1	1	3	9	9B	3.5	0.05
SP11	3	3	2	1	4	1	16		
SP11	3	3	2	1	4	2	17	4	0.0685
SP11	3	3	2	1	4	3	30	2.8	0.0605
SP11	3	3	2	1	4	4	31		
SP11	3	3	2	1	4	5	36	1.75	0.044
SP11	3	3	2	1	4	6	20A	4.4	0.0927
SP11	3	3	2	1	4	7	20B		
SP11	3	3	2	1	4	8	24A	5	0.1018
SP11	3	3	2	1	4	9	24B	3.2	0.0789

experiment	block	trt	CO2	O3	Chamber	SUBREP	flower ID	flower length	flower wt
SP11	3	3	2	1	4	10	24C	2.7	0.0429
SP11	3	3	2	1	4	11	25A		
SP11	3	3	2	1	4	12	25B		
SP11	3	3	2	1	4	13	25C	3	0.0689
SP11	3	3	2	1	4	14	27A	6.4	0.1266
SP11	3	3	2	1	4	15	27B		
SP11	3	3	2	1	4	16	27C	3.1	0.0689
SP11	3	3	2	1	4	17	33A	6.2	0.1378
SP11	3	3	2	1	4	18	33B	4.5	0.1094
SP11	3	3	2	1	4	19	33C	2.1	0.0373
SP11	3	3	2	1	4	20	33D	4.25	0.1248
SP11	3	3	2	1	4	21	33E		
SP11	3	3	2	1	4	22	9A		
SP11	3	3	2	1	4	23	9B		
SP11	3	3	2	1	4	24	9C	2	0.029
SP11	3	3	2	1	4	25	9D	3.25	0.0475
SP11	4	2	1	2	5	1	1	4.6	0.0745
SP11	4	2	1	2	5	2	19	4.6	0.0478
SP11	4	2	1	2	5	3	20	3	0.0547
SP11	4	2	1	2	5	4	22		
SP11	4	2	1	2	5	5	26	3.2	0.0245
SP11	4	2	1	2	5	6	38	4	0.0567
SP11	4	2	1	2	5	7	41	3.7	0.0633
SP11	4	2	1	2	5	8	12A	4.8	0.0644
SP11	4	2	1	2	5	9	12B	3.8	0.0665
SP11	4	2	1	2	5	10	14A	4.3	0.0524
SP11	4	2	1	2	5	11	14B	4	0.0528

experiment	block	trt	CO2	O3	Chamber	SUBREP	flower ID	flower length	flower wt
SP11	4	2	1	2	5	12	14C	2.2	0.0286
SP11	4	2	1	2	5	13	40A	4.5	0.0519
SP11	4	2	1	2	5	14	40B	2.6	0.0482
SP11	4	2	1	2	5	15	40C	2.7	0.0599
SP11	4	2	1	2	5	16	40D	3	0.0511
SP11	4	2	1	2	5	17	40E	2.1	0.0383
SP11	4	3	2	1	6	1	5	3	0.0542
SP11	4	3	2	1	6	2	28	4.5	0.0877
SP11	4	3	2	1	6	3	29		
SP11	4	3	2	1	6	4	37	2.5	0.0511
SP11	4	3	2	1	6	5	25A	2.5	0.0534
SP11	4	3	2	1	6	6	25B		
SP11	4	3	2	1	6	7	27A	4.5	0.0899
SP11	4	3	2	1	6	8	27B	4.6	0.1129
SP11	4	3	2	1	6	9	27C	2.9	0.0622
SP11	4	3	2	1	6	10	27D		
SP11	4	3	2	1	6	11	27E		
SP11	4	3	2	1	6	12	42A		
SP11	4	3	2	1	6	13	42B	3	0.0782
SP11	4	3	2	1	6	14	42C		
SP11	4	1	1	1	7	1	17	0.9	0.0129
SP11	4	1	1	1	7	2	24	2	0.0425
SP11	4	1	1	1	7	3	27		
SP11	4	1	1	1	7	4	46	2.5	0.0419
SP11	4	1	1	1	7	5	9A		
SP11	4	1	1	1	7	6	9B		
SP11	4	4	2	2	8	1	6	2.5	0.053

experiment	block	trt	CO2	O3	Chamber	SUBREP	flower ID	flower length	flower wt
SP11	4	4	2	2	8	2	12	1.9	0.0517
SP11	4	4	2	2	8	3	28	2	0.0541
SP11	4	4	2	2	8	4	42	5	0.0771
SP11	4	4	2	2	8	5	45	4.2	0.0696
SP11	4	4	2	2	8	6	19A		
SP11	4	4	2	2	8	7	19B	3	0.0756
SP11	4	4	2	2	8	8	19C	2.2	0.0428
SP11	4	4	2	2	8	9	29A	4	0.0449
SP11	4	4	2	2	8	10	29B	1.9	0.0399
SP11	4	4	2	2	8	11	39A		
SP11	4	4	2	2	8	12	39B	6.2	0.1069
SP11	4	4	2	2	8	13	3A	4.1	0.0683
SP11	4	4	2	2	8	14	3B	3.6	0.0566
SP11	4	4	2	2	8	15	3C	2.5	0.033
SP11	4	4	2	2	8	16	3D	4.8	0.0666
SP12	5	4	2	2	1	1	1	4.6	0.0589
SP12	5	4	2	2	1	2	2	3.3	0.0638
SP12	5	4	2	2	1	3	3	3.7	0.0863
SP12	5	4	2	2	1	4	4	3.5	0.0478
SP12	5	4	2	2	1	5	5	4.5	0.1367
SP12	5	1	1	1	2	1	1	4.5	0.0707
SP12	5	1	1	1	2	2	2	5.1	0.1379
SP12	5	1	1	1	2	3	3	3	0.0294
SP12	5	1	1	1	2	4	4	5.3	0.0986
SP12	5	1	1	1	2	5	5	4.3	0.0864
SP12	5	3	2	1	3	1	1	5.8	0.1587
SP12	5	3	2	1	3	2	2	4.2	0.0676

experiment	block	trt	CO2	O3	Chamber	SUBREP	flower ID	flower length	flower wt
SP12	5	3	2	1	3	3	3	4.2	0.0613
SP12	5	3	2	1	3	4	4	4.7	0.0793
SP12	5	3	2	1	3	5	5	4.7	0.1006
SP12	5	3	2	1	3	6	6		
SP12	5	3	2	1	3	7	7	5.6	0.1523
SP12	5	3	2	1	3	8	8		
SP12	5	3	2	1	3	9	9	4	0.0717
SP12	6	1	1	1	4	1	1	3.8	0.0839
SP12	6	1	1	1	4	2	2	4.5	0.0586
SP12	6	1	1	1	4	3	3	7.2	0.1138
SP12	6	1	1	1	4	4	4	4.2	0.058
SP12	6	4	2	2	5	1	1	3.5	0.1094
SP12	6	4	2	2	5	2	2	5	0.1186
SP12	6	4	2	2	5	3	3	5.2	0.1261
SP12	6	4	2	2	5	4	4	4.8	0.0642
SP12	6	4	2	2	5	5	5	4.3	0.0818
SP12	5	2	1	2	6	1			
SP12	6	3	2	1	7	1	1		
SP12	6	3	2	1	7	2	2		
SP12	6	3	2	1	7	3	3	3.1	0.0363
SP12	6	3	2	1	7	4	4	6	0.1347
SP12	5	2	1	2	8	1	1	7.3	0.1174
SP12	5	2	1	2	8	2	2	3	0.0476

Phleum pratense L.

treatments

								flower length	flower wt
experiment	block	trt	CO2	O3	Chamber	SUBREP	flower ID		
	ambient (1)	elevated (2)			trt 1	trt 2	trt 3		
CO2	400 ppm	800 ppm			30 ppb O3	80 ppb O3	30 ppb O3		
O3	30 ppb	80 ppb			400 ppm CO2	400 ppm C	800 ppm C		
3 experiment	FA11	SP11	SP12						
8 chambers per experiment in a complete factorial design									
2 reps (block) per experiment									
Chamber experiment (CSTR)									
All flowers collected from each chamber									
flower bagged upon emergence and immediately removed and placed in freezer after dehiscence of pc									
Flowers washed with PBS-T (3 washes; pollen removed from each wash via centrifuge; three washes cc									
pollen suspended in known volume and counted using hemocytometer (the total amount of pollen fror									
Phl-p-5 content determined using ELISA									
ALL FLOWERS WERE COLLECTED FROM ALL CHAMBERS									
Some flowers were damaged in collection (ie pollen lost) and not used in analysis of pollen content or p									
